# Is resistance training effective to increase BDNF in multiple sclerosis? An updated systematic review and meta-analysis of isolated interventions

**DOI:** 10.1371/journal.pone.0359019

**Published:** 2026-10-27

**Authors:** William Riciere Pedon, Gustavo De Conti Teixeira Costa, Lorenzo de Ávila Rodrigues Cortizo Vidal, Pedro Augusto Inacio, Marcelo Magalhães Sales, Ivanéria Mendonça Martins, James Oluwagbamigbe Fajemiroye, Sergio Machado, Vicente Aprigliano, Gabriela Ribeiro Inacio, Adolfo Mundombe Cameia, Gaspar R. Chiappa, Eduardo da Matta Mello Portugal, Alberto Souza Sá Filho

**Affiliations:** 1 Graduate Program in Human Movement and Rehabilitation, Evangelical University of Goiás (UniEVANGÉLICA), Anápolis, Goiás, Brazil; 2 Faculdade de Educação Física e Dança – FEFD, Universidade Federal de Goiás (UFG – Campus Samambaia), Goiânia, Goiás, Brazil; 3 Graduate Program in Environmental and Society of State University of Goiás (UEG), Academic institute of health and biological sciences. Southwest Campus, Quirinópolis, Goiás, Brazil; 4 Graduate Program in Pharmaceutical Sciences, Pharmacology and Therapeutics, Evangelical University of Goiás (UniEVANGÉLICA), Anápolis, Goiás, Brazil; 5 Institute of Biological Sciences, Federal University of Goiás, Goiânia, Goiás, Brazil; 6 Graduate Program in Mental Health and Psychiatry; Federal University of Rio de Janeiro, UFRJ-IPUB, Rio de Janeiro, Brazil; 7 Escuela de Ingeniería de Construcción y Transporte, Pontificia Universidad Católica de Valparaíso, Valparaíso, Chile; 8 Departamento de Pediatria da Universidade Rainha Njinga a Mbande (URNM), Malanje, Angola; 9 Faculty of Health Sciences, Universidad Autónoma de Chile, Providencia, Chile; 10 Graduate Program in Physical Education at Federal University of Rio de Janeiro, UFRJ, Rio de Janeiro, Brazil; University of Rijeka Faculty of Health Studies: Sveuciliste u Rijeci Fakultet zdravstvenih studija, CROATIA

## Abstract

**Background:**

Brain-derived neurotrophic factor (BDNF) is a key mediator of neuroplasticity and has been associated with disease progression in multiple sclerosis (MS). While aerobic exercise is consistently linked to BDNF modulation, the specific contribution of isolated resistance training (RT) remains unclear, as previous reviews have largely combined multimodal exercise interventions.

**Objective:**

To systematically review and meta-analyze the effects of isolated RT on peripheral BDNF levels in individuals with MS (PROSPERO: CRD420251141069).

**Methods:**

Searches were conducted in PubMed, Web of Science, Scielo, Scopus, PEDro and Cochrane up to January 10, 2026, using PICOT criteria. Controlled experimental studies investigating isolated RT and BDNF outcomes were included. Risk of bias was assessed using RoB 2.0 and ROBINS-I, and certainty of evidence was evaluated using GRADE. Due to methodological heterogeneity, a random-effects model was applied to estimate pooled effects.

**Results:**

Four studies (three RCTs and one controlled non-randomized trial), totaling 126 participants with MS, were included. Interventions lasted 8–24 weeks, with training frequencies of 2–3 sessions per week. Meta-analytic synthesis revealed positive pooled effects (SMD = 0.49; 95%CI: 0.15 to 0.83; Z = 2.80; p < 0.005; I^2^  = 0%; Q = 1.681) with wide confidence intervals without crossing zero. With the Hartung–Knapp adjustment, due to the reduced number of studies, the analysis continued to show a positive intervention effect: (SMD = 0.49; 95%CI: 0.42 to 0.56; t = 3.716; p = 0.0339). Two studies using low-to-moderate intensity protocols (Pilates-based RT and low-load RT with bands/weights) showed significant increases in serum BDNF after 8 weeks. In contrast, studies employing higher loads (≈80% of one-repetition maximum: 1RM) demonstrated no significant BDNF changes despite functional gains. Overall certainty of evidence was rated as low due to risk of bias, inconsistency, and imprecision.

**Conclusion:**

Isolated RT may increase peripheral BDNF levels in individuals with MS, but this effect is supported by limited and low-certainty evidence. Furthermore, given that only four studies were included in the meta-analysis, we suggest interpreting the results with some caution.

## Introduction

Multiple sclerosis (MS) is a chronic immune-mediated and neurodegenerative disease of the central nervous system characterized by demyelination, axonal injury, and progressive neurological disability [1–3]. Beyond inflammatory activity, MS is associated with impaired neuroplasticity, motor dysfunction, fatigue, cognitive impairment, and reduced functional capacity, which substantially affect patients’ quality of life [4–6]. Although disease-modifying therapies have improved the control of inflammatory disease activity, non-pharmacological strategies capable of supporting neuroprotection and functional recovery remain clinically relevant [7–9].

Brain-derived neurotrophic factor (BDNF) is a key neurotrophin involved in neuronal survival [10–12], synaptic plasticity [13], axonal repair [14], and adaptive responses to neurological injury [15]. Reduced circulating BDNF levels have been reported in individuals with MS and may be associated with disease severity and progression [16,17]. Therefore, interventions capable of modulating BDNF have gained attention as potential adjunctive strategies in MS rehabilitation [9,12,17].

Physical exercise is one of the most studied non-pharmacological interventions for promoting neuroplastic and functional adaptations in neurological populations [5,7,11,12,15,18,19]. In MS, aerobic and combined exercise programs have been associated with improvements in mobility, fatigue, cognition, and selected neurobiological markers [8,20–22]. However, the specific contribution of resistance training (RT) remains less clear. This seems paradoxical, given that RT has been strongly and widely recommended in MS rehabilitation due to its effects on muscle strength, functional independence, fatigue, and mobility [23,24]. It is worth noting that both aerobic and resistance exercises are recommended by the position statement on exercise for people with mild to moderate multiple sclerosis from Exercise & Sports Science Australia (ESSA) [25].

Current evidence on exercise-induced BDNF responses in MS is limited by the frequent combination of different exercise modalities, including aerobic, balance, functional, and resistance-based interventions [26–30]. As a result, it remains difficult to determine whether RT alone is sufficient to modify peripheral BDNF levels. Moreover, available studies differ substantially in training intensity, disease phenotype, disability level, and biological matrix used for BDNF quantification, particularly serum versus plasma, which may influence the interpretation of findings [31–34].

Therefore, the present systematic review and meta-analysis aimed to investigate the effects of isolated RT on peripheral BDNF levels in individuals with MS. By focusing exclusively on resistance-based interventions, this review seeks to clarify whether RT has an independent neurotrophic effect and to identify methodological factors that may explain variability among studies.

## Methods

The present study followed the standard recommendations of the Cochrane Handbook for Systematic Reviews of Interventions and the Preferred Reporting Items for Systematic Reviews and Meta-Analyses (PRISMA) guidelines [35,36]. This review was registered in the International Prospective Register of Systematic Reviews (PROSPERO; registration number: CRD420251141069). The completed PRISMA 2020 checklist is available in the in S1 File (Table S1).

### Search strategy and databases

A systematic search for relevant studies was conducted in PubMed/MEDLINE, ISI Web of Science, SciELO, Scopus, PEDro and the Cochrane Library by combining free-text terms and MeSH descriptors, using a broad range of synonyms and related terms according to the PICOT framework:

P = Participants with multiple sclerosis (“multiple sclerosis” OR “sclerosis, multiple”);

I = Resistance training or strength training (“resistance training” OR “strength training”);

C = No exercise or traditional rehabilitation;

O = BDNF or brain-derived neurotrophic factor (“BDNF” OR “brain-derived neurotrophic factor”);

T = Randomized controlled trials or controlled experimental studies, including parallel quasi-experimental designs.

The systematic literature search was carried out without restrictions on language or publication date, aiming to identify studies that specifically investigated the effects of isolated RT on BDNF levels in patients with MS (the complete strategy can be viewed in the supplementary S2 File). Therefore, the guiding research question was: Can RT modify BDNF levels in individuals with MS?

The search strategy combined structured terms as follows: “Multiple Sclerosis” AND (“Resistance Training” OR “Strength Training”) AND (“BDNF” OR “brain-derived neurotrophic factor”). Search combinations were adapted according to the indexed descriptors and specific requirements of each database, as presented in S1 File (Table S2).

Searches were conducted up to January 10, 2026. In addition, the reference lists of the included studies were manually screened to identify potentially eligible records. To minimize the risk of missing relevant studies, additional searches were carried out in the grey literature, including generic web searches, and in the reference lists of previous reviews.

Two authors independently and thoroughly screened potentially relevant articles using the eligibility criteria defined according to the PICOT framework.

### Inclusion and exclusion criteria

Only articles published in peer-reviewed journals reporting results from controlled experimental studies or randomized controlled trials were included. Considering the limited number of studies available on this topic before the beginning of the review, articles published in any language were considered eligible.

Unpublished studies, preprints, conference posters, and studies reporting non-experimental designs were excluded. Non-experimental studies included, for example, uncontrolled pre–post intervention studies and case series.

### Study selection and data extraction

Study selection was carried out in two stages by two independent reviewers. First, the titles and abstracts of records identified in the databases were screened to exclude clearly irrelevant studies. After that, the full texts of potentially eligible articles were retrieved and assessed according to the predefined inclusion and exclusion criteria. Disagreements between reviewers were resolved during a consensus meeting and, when necessary, by consultation with a third reviewer.

After the final selection of included studies, data extraction was independently carried out by two reviewers using a standardized spreadsheet specifically developed for this review. The following information was extracted: author and year of publication, country where the study was conducted, sample characteristics, sample size, sex, age, MS subtype, disease duration, Expanded Disability Status Scale (EDSS) score, study design, control group characteristics, details of the RT intervention, type of exercise, program duration, weekly frequency, intensity, volume, progression, BDNF assessment method, biological matrix, laboratory technique, time point of blood collection, outcomes assessed, main pre- and post-intervention results, and information required for quantitative synthesis.

When available, mean values, standard deviations, and sample sizes for the intervention and control groups were also collected to allow calculation of effect sizes for the meta-analysis. Two authors independently extracted and reviewed the data, and each extraction was checked by another author. WebPlotDigitizer was used to extract numerical data from figures when necessary. In cases where data were incomplete, ambiguous, or presented only in graphical format, extraction was performed based on the information available in the article, prioritizing complete and clinically comparable data across studies. Any disagreements during data extraction were resolved by consensus among the reviewers.

### Risk of bias assessment

The risk of bias of the included studies was independently assessed by two reviewers using the appropriate Cochrane tools according to each study design. For randomized controlled trials, the revised Cochrane Risk of Bias tool (RoB 2) was applied [37]. This tool evaluates five domains: the randomization process, deviations from the intended interventions, missing outcome data, outcome measurement, and selection of the reported results. Each domain was classified as “low risk,” “some concerns,” or “high risk,” and an overall judgment was then assigned based on these assessments.

For non-randomized intervention studies, the Risk of Bias in Non-randomized Studies of Interventions tool (ROBINS-I) was used. This instrument assesses seven domains: bias due to confounding, selection of participants, classification of interventions, deviations from intended interventions, missing data, measurement of outcomes, and selection of the reported results. Each domain was classified as having “low,” “moderate,” “serious,” or “critical” risk of bias, with the overall judgment corresponding to the highest level of risk identified across domains.

Disagreements between reviewers were resolved during a consensus meeting and, when necessary, with the involvement of a third reviewer. The results of the risk of bias assessment were summarized narratively and graphically for each study.

### Certainty of evidence

The quality, or strength, of the evidence from the included studies was assessed using the Grading of Recommendations Assessment, Development and Evaluation (GRADE) approach, according to the recommendations of the GRADE Working Group [38]. This framework considers five main domains: risk of bias, inconsistency, indirectness, imprecision, and publication bias, allowing the certainty of evidence to be classified as high, moderate, low, or very low.

All eligible randomized controlled trials were initially rated as high-certainty evidence. The final rating was then downgraded according to the methodological limitations identified across the five GRADE domains. The assessment was conducted independently by two reviewers, and disagreements were resolved by consensus.

### Statistical analysis

The quantitative synthesis was conducted through meta-analysis using a random-effects model, considering the methodological and clinical variability among the included studies. Effect sizes were expressed as standardized mean differences (SMDs) with their respective 95% confidence intervals (95% CIs), using Hedges’ correction for small samples.

For studies reporting continuous data, mean values, standard deviations, and sample sizes were extracted for the intervention and control groups. When necessary, effect sizes were calculated from pre- to post-intervention differences between groups. In cases where data were incomplete or presented only in graphical format, values were estimated based on the information available in the articles.

The meta-analysis was carried out using the restricted maximum likelihood (REML) estimator to estimate between-study variance (τ^2^), together with the Hartung–Knapp adjustment (*Student’s t* – distribution), which is considered more robust for meta-analyses including a small number of studies. The Hartung–Knapp adjustment was applied to account for statistical uncertainty, particularly when few studies are involved [39]. This adjustment involves modifying the reference distribution by replacing the critical value (*Z*_1−α/2_) with the critical value (*t*_1−α/2; k−1_) and setting the degrees of freedom to (*k* − 1), where *k* is the total number of studies included in the meta-analysis. Additionally, the standard error is adjusted by multiplying the standard error by a correction factor (*q*), calculated based on Cochrane’s *Q* statistic and the study weights.

Heterogeneity among studies was assessed using Cochran’s Q test and quantified using the I^2^ statistic, interpreted according to the following thresholds: low heterogeneity, 25%; moderate heterogeneity, 50%; and high heterogeneity, 75%. Between-study variance (τ^2^) was also reported as a complementary measure of heterogeneity.

The statistical significance of the overall effect was determined using the Z test. In the adjusted analysis, the critical Z-value was replaced by the critical value of the Student’s t-distribution, and the degrees of freedom were defined as (k − 1), where k is the total number of studies included in the meta-analysis [40]. The significance level was set at 5% (p < 0.05). The direction of effects was standardized so that positive SMD values indicated a benefit of the intervention compared to the control group.

Diagnostic sensitivity analyses were conducted using the leave-one-out approach to assess the presence of influential studies and outliers. Publication bias was evaluated through visual inspection of the funnel plot and Egger’s regression test. In addition, the trim-and-fill method was applied to estimate the number of potentially missing studies and their impact on the pooled effect size. The number of studies required to nullify the statistical significance of the findings was estimated using the fail-safe N. All analyses were performed using Jamovi statistical software, version 2.7.18, with the significance level set at 5% (p < 0.05).

## Results

A comprehensive systematic search was conducted across PubMed/MEDLINE, Web of Science, Scopus, Cochrane Library, SciELO, and PEDro, yielding a total of 15,848 records. After applying database-specific filters, 232 records were retained from PubMed, 252 from Web of Science, 378 from Scopus, 206 from the Cochrane Library, and 4 from PEDro, while no relevant studies were identified in SciELO. Following the consolidation of all records into a single database, 1,072 studies were subjected to initial title screening. Duplicate removal was carried out using a combination of automated and manual procedures, resulting in 931 unique records after the exclusion of 141 duplicates.

Subsequently, studies were screened based on relevance to the target population, restricting eligibility to individuals diagnosed with MS, which reduced the number of potentially relevant records to 300. Further screening based on the primary outcome of interest (BDNF levels) led to the exclusion of 272 studies, leaving 28 articles eligible for full-text assessment. Of these, 23 studies were excluded for not meeting the predefined inclusion criteria, primarily due to the absence of isolated RT interventions or lack of BDNF-related outcomes. Additionally, one study was identified as a duplicate publication reported under different titles and was therefore excluded.

Ultimately, four studies met all eligibility criteria and were included in the qualitative and quantitative synthesis. Among these, three were randomized controlled trials and one was a non-randomized controlled study with a within-subject design. The included studies investigated exclusively RT interventions, employing modalities such as free weights, elastic bands, and Pilates-based resistance exercise. S1 File (Table S1) presents the search strategy, with the respective access links and the database from which the articles were extracted. The studies excluded after full-text assessment, along with the corresponding reasons for exclusion, are presented in Supplementary Table S3 in S1 File. Fig 1 presents the data extraction and final inclusion of articles for meta-analysis.

**Fig 1:**
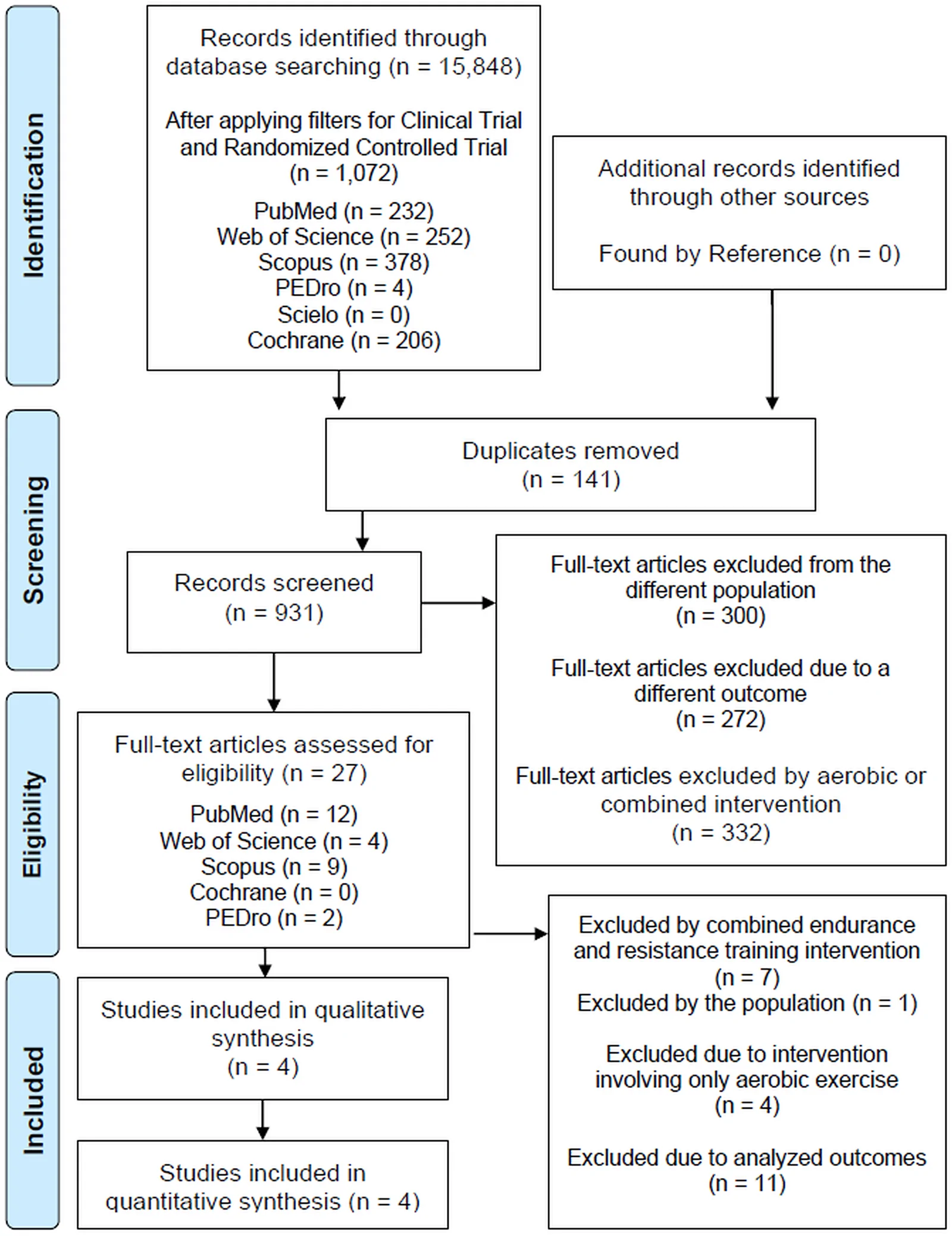
PRISMA flow diagram.

### Characteristics of the studies

The four included studies were published between 2014 and 2025. The included studies were conducted in different geographical settings, comprising populations from Europe and the West Asia, specifically Denmark, the Netherlands, and Iran. Three studies were published in English, and one study was published in Persian, being translated through artificial intelligence (Chat GPT version 5.2).

Among the included studies, a total of 126 participants with MS were evaluated. The samples were predominantly composed of women, with one study including exclusively female participants, while the others included mixed-sex samples with a female predominance. Participant age varied substantially among studies, ranging from younger adults (± 33 years) to older individuals with progressive disease (± 54 years), reflecting variability in disease stage and clinical profiles.

Regarding clinical characteristics, most participants were diagnosed with relapsing-remitting MS, although one study specifically included individuals with progressive MS, characterized by longer disease duration (approximately 20 years) and higher disability levels. Disability status, assessed using the EDSS, ranged from mild to moderate impairment (EDSS 2–6) in relapsing-remitting populations to moderate disability (median EDSS ≈4.5) in progressive cases, indicating heterogeneity in disease severity across the included samples.

The intervention models also varied considerably in terms of structure, intensity, and training objectives. All studies investigated resistance-based exercise modalities; however, the specific protocols ranged from low-to-moderate intensity programs, such as Pilates focusing on core stability, balance, and controlled movements, to high-intensity progressive RT protocols involving machine-based and free-weight exercises targeting major muscle groups. Intervention duration ranged from 8 to 24 weeks, with training frequencies between two and three sessions per week. Lower-intensity interventions emphasized gradual progression and patient tolerance, whereas higher-intensity protocols applied structured load progression (e.g., from ≈15 repetition maximum to ≈6 repetition maximum) and supervised training sessions. It is worth noting that, in all studies eligible for inclusion in the present study, individuals in the control group did not receive any type of intervention.

The included studies represent a heterogeneous set of populations and intervention approaches, encompassing different stages of MS, varying levels of disability, and a wide spectrum of RT modalities, from rehabilitative low-load exercise to high-intensity strength training. Table 1 presents the sample description and results.

**Table 1:** Summary of findings on the effects of RT on BDNF levels in patients.

Author (year)	Design/ Sample	Characteristics	Intervention	BDNF evaluated	Effect	Outcome/Results
Eftekhari & Etemadifar [31]	RCT; n = 29 women with relapsing-remitting MS (15 Pilates/ 14 Control after losses);	Age: 33 ± 8.08 years; BMI: 24.52 ± 4.93 kg/m^2^; EDSS 2–6	Mat Pilates (3 times/week for 8 weeks); progressive light to moderate intensity.	Serum BDNF Enzyme-Linked immunosorbent assay (ELISA); pre- and post-8 weeks	Significant increase for Pilates; (F = 5.12; p = 0.03; η = 0.18)	↑ BDNF (10678.5 ± 2260.1 to 11550.1 ± 2619.60 vs. 11377.4 ± 3860.6 ng/ml), respectively for RT and control; ↔ No change in IL-10 (13.1 ± 5.4 to 12.3 ± 7.2 vs. 13.2 ± 4.76 to 11.7 ± 4.8 ng/ml), respectively for RT and control.
Jørgensen et al. [33]	RCT; n = 30 with remitting MS (16 RT/ 14 Control); 24 weeks	RT – Age: 44 [40:51] years, EDSS score 3.0 [2.0:3.5]; Control – 45 [37:47] years, EDSS score 3.0 [2.5:3.5]	Progressive RT (2x/week, ≈ 80% 1RM)	Plasma BDNF ELISA; in acute and chronic analysis (pre/post intervention	No significant change	BDNF at rest Pre vs. Post: RT – Median [IQR] 142.0 [83.8:274.3] ↔158.5 [83.7:218.8] and Control - ↔ 167.7 [48.2:296.2] 99.3 [86.5:210.7].
Moghadasi et al. [34]	Pilot RCT; n = 27 women (14 intervention/ 13 control); mild to moderate MS	Age 32.4 ± 5.5 years and EDSS score between 1 and 5.	RT with weights/resistance bands (3 times/week for 8 weeks); 50% to 65% of one repetition maximum.	Serum BDNF ELISA (pre- and post-intervention)	Significant increase in the intervention group (27.5%; p = 0.04)	Pre- and post-treatment BDNF: RT – 1068.4 to 1362.7 pg/mL; EDSS reduction (2.1 ± 1.4 to 1.3 ± 1.5; ↓ 27.7%); Fat: 23.2 ± 8.5 kg to 22.3 ± 8.5 kg (↓5.1%);
Gravesteijn et al. [32]	CT; 16 weeks of Self-Control vs. 16 weeks of Intervention vs. 16 weeks of Follow-up; n = 30 (25 women)	Progressive MS, mean age 54 years, EDSS 4.5 [4.0–5.5], disease duration: 20.2 ± 10.1 years	Progressive RT for 16 weeks, 3 times a week, with physiotherapy supervision.	Plasm BDNF (high-sensitivity multiplex immunoassay kit)	No significant difference; upward trend: + 9%	BDNF: baseline 1 and baseline 2 (7% increase, 95% CI: −19% to +41%), between baseline 2 and post-intervention (9% increase, 95% CI: −18% to +44%), and between post-intervention and follow-up (3% increase, 95% CI: −22% to +37%).

RT = resistance training; CT = controlled trial; RCT = randomized controlled trial; MS = multiple sclerosis; BMI = body mass index; Median [IQR] = median ± interquartile range.

### Summary of findings

A total of four studies (k = 4) were included in the quantitative synthesis evaluating the effects of the intervention on BDNF levels. The meta-analysis was conducted using a random-effects model with the restricted maximum-likelihood estimator (REML) and Hartung–Knapp adjustment, accounting for potential between-study variability.

The pooled analysis demonstrated a statistically significant positive effect of the intervention on BDNF levels, with a standardized mean difference (SMD) of 0.492 (SE = 0.131), corresponding to a 95% confidence interval ranging from 0.15 to 0.83 (Z = 2.80, p = 0.0050). This result indicates a moderate effect size, favoring the intervention. As illustrated in the forest plot (Fig 2), all included studies showed effect estimates in the positive direction, with individual SMDs ranging from 0.27 to 0.90. Although only one study (Moghadasi et al.[34],) demonstrated a statistically significant individual effect, the consistency in the direction of effects across studies supports the robustness of the pooled estimate. In addition, with the Hartung–Knapp adjustment, due to the reduced number of studies [39], the analysis continued to show a positive intervention effect: (SMD = 0.49; 95%CI: 0.42 to 0.56; t = 3.716; p = 0.0339)

**Fig 2:**
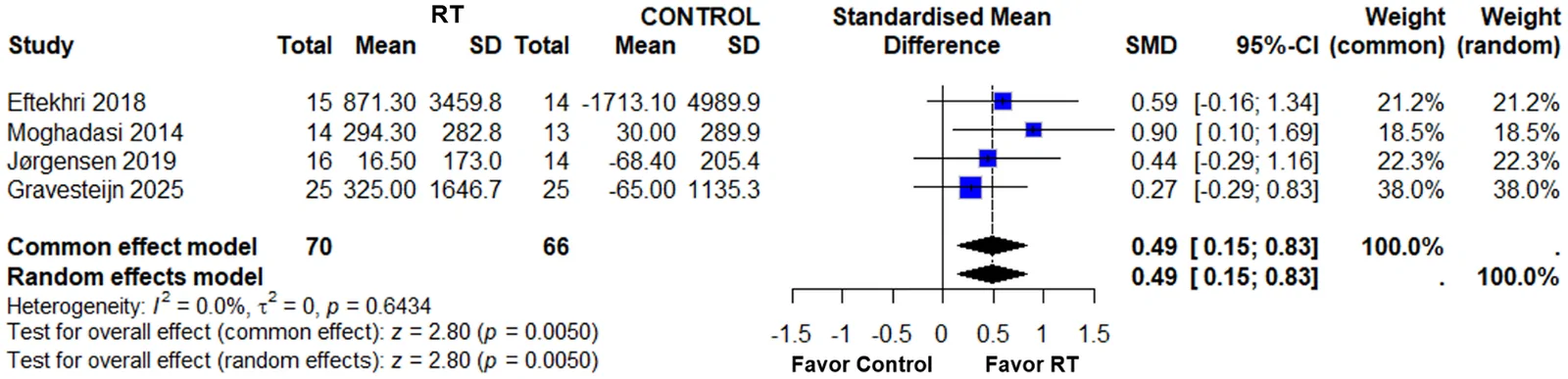
Forest plot of the effects of the intervention on BDNF levels. Squares represent individual study effect sizes, with their size proportional to the study weight, and horizontal lines indicate 95% confidence intervals. The diamond at the bottom represents the overall pooled effect size and its confidence interval.

Regarding heterogeneity, no significant between-study variability was observed. The Cochran’s Q-test indicated non-significant heterogeneity (Q(3) = 1.681, p = 0.643), and the I^2^ statistic was 0%, with a between-study variance estimate (τ^2^) of 0.000. These findings suggest that the observed variability in effect sizes is likely attributable to sampling error rather than true differences between studies, indicating a high degree of consistency across the included evidence.

Leave-one-out analysis identified the study by Moghadasi et al. [34] as an influential/outlying study. Therefore, when it was removed from the analysis, a reduction in the combined effect was observed. Fig 3 shows the results of the sensitivity analysis. Despite the change in the combined effect, the outcome for BDNF was not modified.

**Fig 3:**
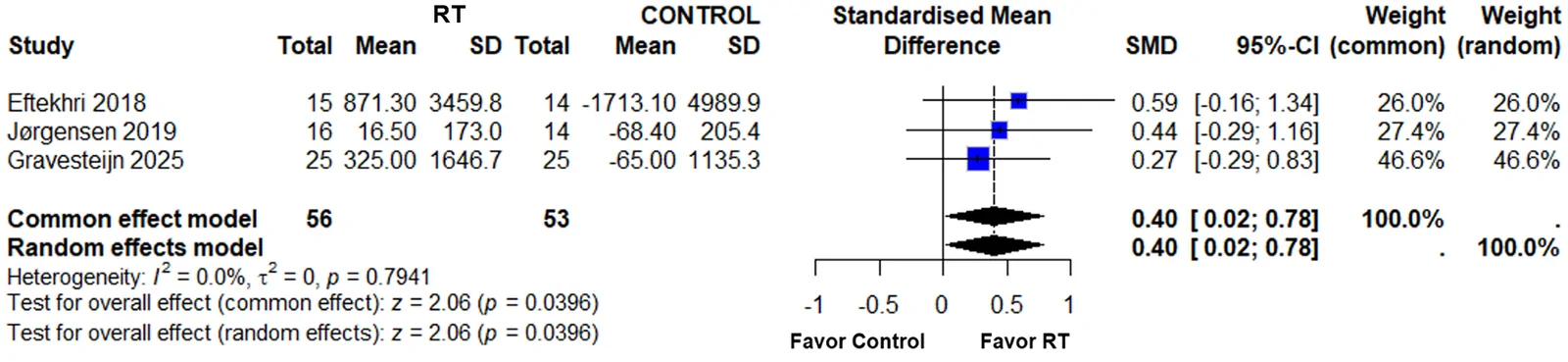
Forest plot from sensitivity analysis on BDNF levels. Squares represent individual study effect sizes, with their size proportional to the study weight, and horizontal lines indicate 95% confidence intervals. The diamond at the bottom represents the overall pooled effect size and its confidence interval.

Publication bias was assessed through multiple complementary approaches. Given fewer than 10 studies, these analyses are exploratory. Neither Egger’s regression test (p = 0.115) nor Begg and Mazumdar’s rank correlation test (p = 0.083) indicated statistically significant asymmetry. However, the fail-safe N was calculated as 9, suggesting that a relatively small number of additional null-effect studies would be sufficient to attenuate the observed significance. In addition, the trim-and-fill method estimated that two potentially missing studies could exist, likely on the left side of the distribution.

Visual inspection of the funnel plot (Fig 4) revealed a slight asymmetry, characterized by a relative absence of studies with smaller or negative effects. Although this pattern may suggest the presence of small-study effects or potential publication bias, the limited number of included studies reduces the interpretability and statistical power of such analyses. Therefore, these findings should be interpreted with some caution.

**Fig 4:**
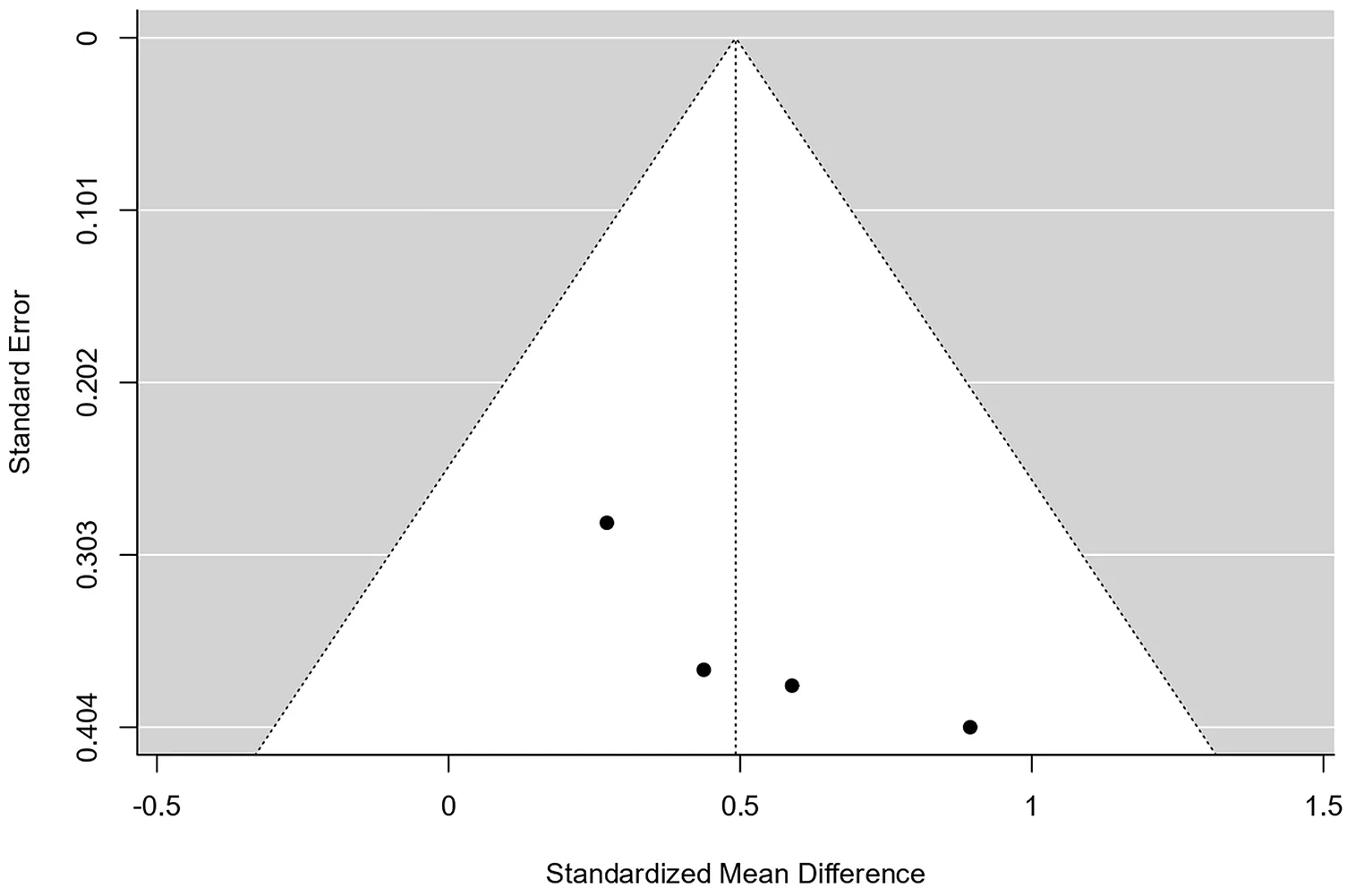
Funnel plot for publication bias assessment of the included studies. The vertical dashed line represents the pooled effect size, while the diagonal dashed lines indicate the expected 95% confidence limits in the absence of publication bias. Each dot corresponds to an individual study. A symmetric distribution around the pooled effect suggests low risk of publication bias, whereas asymmetry may indicate small-study effects or potential publication bias.

### Determining the risk of bias

Risk of bias was assessed using the revised Cochrane Risk of Bias tool (RoB 2) for randomized controlled trials and the ROBINS-I tool for the non-randomized intervention study, in accordance with Cochrane Handbook recommendations.

Among the included randomized controlled trials, all three studies were judged as presenting some concerns for overall risk of bias. In the study by Jørgensen et al. [33], the randomization process was adequately described, including concealed allocation and stratification by sex, suggesting low risk of bias in this domain. However, the present analysis was based on secondary exploratory, per-protocol analyses of blood samples, and a proportion of participants were excluded after randomization due to missing biological data. These factors raise concerns regarding the domains of missing outcome data and selection of the reported result, particularly in the absence of a clearly reported prespecified analysis plan for the BDNF outcome. Outcome measurement was performed using standardized enzyme-linked immunosorbent assay (ELISA) procedures, which supports a low risk of bias in the measurement domain.

In the study by Eftekhari et al. [41], participants were randomized using sealed envelopes; however, insufficient details were provided regarding sequence generation and allocation concealment procedures, leading to some concerns in the randomization domain. Additionally, participant attrition was reported without comprehensive clarification of group-specific losses, contributing to potential bias in the missing outcome data domain. Although outcome assessment was based on objective laboratory measurements (ELISA), reducing the likelihood of measurement bias, the absence of a registered protocol or prespecified statistical analysis plan resulted in some concerns regarding selective reporting.

Similarly, in the study by Moghadasi et al. [42], although random allocation to intervention and control groups was reported, methodological details regarding the randomization process, allocation concealment, and blinding were insufficiently described. While the objective measurement of BDNF using laboratory methods supports low risk in the outcome measurement domain, the lack of transparency in key methodological aspects and absence of protocol registration led to an overall judgment of some concerns.

The study by Gravesteijn et al. [32] was evaluated using the ROBINS-I tool, as it employed a non-randomized, within-person design with an extended baseline period. This study was judged as having a moderate risk of bias, primarily due to potential confounding and the absence of randomized allocation. Although the study was prospectively registered and employed appropriate statistical adjustments for key confounders (e.g., age, sex, and EDSS), inherent limitations of the design remain. Outcome assessment was conducted using objective biomarker analyses with advanced techniques (high-sensitivity multiplex immunoassay kit), supporting low risk in the measurement domain, and reporting appeared consistent with the study protocol.

### Analysis of the strength of evidence

The certainty of evidence for the effect of RT on peripheral BDNF levels in individuals with MS was assessed using the GRADE framework. Although the body of evidence included randomized controlled trials, several methodological limitations led to downgrading. First, the risk of bias was judged as serious, as most studies presented concerns related to insufficient reporting of randomization procedures, lack of allocation concealment details, and absence of prespecified analysis plans. Additionally, small sample sizes and incomplete reporting contributed to uncertainty in the internal validity of the included studies.

Inconsistency across studies was considered moderate but not sufficient for full downgrading, as effect estimates were consistently in the same direction (favoring RT), despite differences in statistical significance. However, variability in intervention characteristics (e.g., intensity, duration, and training modality), participant profiles (relapsing-remitting vs. progressive MS), and biological matrices used for BDNF assessment (serum vs. plasma) suggests underlying clinical and methodological heterogeneity.

Indirectness was judged as not serious, as all included studies directly addressed the research question, evaluating isolated RT interventions and their effects on peripheral BDNF levels in individuals with MS.

Imprecision was considered serious, primarily due to small sample sizes across all included studies and relatively wide confidence intervals around individual effect estimates. Although the pooled effect did not cross the null, the total sample size did not meet the Optimal Information Size, limiting confidence in the precision of the estimate.

Potential publication bias could not be definitively ruled out due to the small number of included studies. While statistical tests did not indicate significant asymmetry, the presence of small-sample studies with predominantly positive findings raises concern for small-study effects.

These limitations resulted in an overall low certainty of evidence, indicating that further high-quality randomized trials are likely to have an important impact on the confidence in the estimate and may change the magnitude or direction of the observed effect. Table 2 presents a summary of the GRADE.

**Table 2:** GRADE summary of findings.

Outcome	Studies Included	No. of Studies	n	Effect Size (SMD)	95% Confidence Interval	Risk of Bias	Inconsistency	Indirectness	Imprecision	Certainty of Evidence (GRADE)
Change in BDNF levels	Eftekhari [31]; Moghadasi [34]; Jørgensen [33]; Gravesteijn [32];	4	126	0.49	0.15–0.83	Serious	Moderate	Not serious	Serious	⊕⊕⊝⊝ Low

## Discussion

The present systematic review and meta-analysis suggests that isolated RT may increase peripheral BDNF levels in individuals with MS. The pooled analysis showed a positive and statistically significant effect favoring RT, with a standardized mean difference of moderate magnitude (SMD = 0.492; SE = 0.131; 95% CI: 0.076 to 0.909; Z = 3.76; p = 0.033). In addition, heterogeneity was negligible, as indicated by a non-significant Cochran’s Q test (Q[3] = 1.681; p = 0.643), an I^2^ of 0%, and τ^2^ = 0.000. These findings suggest that the observed effect was directionally consistent among studies, although the small number of included trials requires cautious interpretation.

The main contribution of this review is the isolation of RT as the intervention of interest. Previous meta-analytic evidence has suggested that exercise can increase peripheral BDNF levels in people with MS, but most available syntheses have combined aerobic, resistance, balance, functional, and multimodal interventions, limiting the ability to identify the independent contribution of RT [9,17,43]. In contrast, the present review focused specifically on isolated resistance-based protocols. Therefore, our findings suggest that RT may have an independent neurotrophic effect in this population [31–34]. However, this effect should not be interpreted as definitive, since the evidence is based on only four studies, small samples, and relevant methodological variability in training prescription, clinical phenotype, and BDNF assessment.

The relevance of BDNF in MS is supported by evidence showing that patients with MS tend to present lower circulating BDNF levels than healthy controls. Naegelin et al. [16] reported lower serum BDNF concentrations in patients with MS compared with healthy controls, with values of 29.41 ± 7.24 versus 32.69 ± 8.33, respectively. In that study, clinical severity also differed among disease phenotypes, with EDSS scores increasing from 1.41 ± 0.80 in clinically isolated syndrome to approximately 5.0 in progressive forms of MS. Moreover, the lowest serum BDNF values were observed in patients with secondary progressive MS (27.87 ± 7.55), suggesting that lower BDNF availability may accompany more advanced disease stages [16]. Similarly, Karimi et al. [17], in a meta-analysis including 13 studies, found significantly reduced BDNF levels in people with MS compared with healthy controls, with subgroup effects observed for both serum BDNF (SMD = −0.968; 95% CI: −1.609 to −0.327; p < 0.0001) and plasma BDNF (SMD = −5.199; 95% CI: −8.449 to −1.950; p < 0.0001).

In this context, the positive pooled effect observed in the present review suggests that RT may contribute to a more favorable neurobiological environment in MS. Exercise-induced muscle contraction may activate pathways associated with neuroplasticity, including the PGC-1α/FNDC5/irisin axis, which has been linked to increased BDNF expression in experimental models [11,15,44]. In addition, exercise may influence BDNF through lactate signaling [11,45,46], myokine release [47–49], mitochondrial adaptations, and anti-inflammatory effects, creating a systemic environment that may support neuronal survival and synaptic plasticity [50,51]. Nevertheless, peripheral BDNF should not be interpreted as a direct marker of central neuroplasticity, but rather as an accessible biomarker that may reflect part of the systemic response to exercise.

A relevant pattern observed among the included studies was the apparent influence of training intensity on BDNF responses. The studies that reported significant increases in BDNF used low-to-moderate intensity interventions. Eftekhari and Etemadifar [31] applied Mat Pilates three times per week for eight weeks in women with relapsing-remitting MS and EDSS between 2 and 6. This intervention significantly increased serum BDNF (F = 5.12; p = 0.03; η = 0.18), with values increasing from 10,678.5 ± 2,260.1 to 11,550.1 ± 2,619.6 ng/mL in the intervention group. Similarly, Moghadasi et al. [34] used RT with weights and elastic bands at 50–65% of 1RM, three times per week during eight weeks, and reported a 27.5% increase in serum BDNF (p = 0.04), from 1,068.4 to 1,362.7 pg/mL. These two studies suggest that moderate and clinically tolerable resistance-based stimuli may be sufficient to increase serum BDNF in individuals with MS.

In contrast, studies using higher-intensity progressive RT did not observe significant BDNF changes. Jørgensen et al. [33] investigated progressive RT performed twice per week for 24 weeks at approximately 80% of 1RM in people with relapsing-remitting MS. Despite the structured and prolonged intervention, plasma BDNF did not significantly change, with resting values in the RT group varying from (Median[IQR]) 142.0 [83.8–274.3] to 158.5 [83.7–218.8]. Gravesteijn et al. [32], in individuals with progressive MS, also reported no significant BDNF response after 16 weeks of progressive RT performed three times per week, although an upward trend was observed between baseline and post-intervention (+9%; 95% CI: −18% to +44%).

This pattern contrasts with evidence obtained in non-MS populations. For example, Borges Junior et al. [52] reported that a single RT session at 80% of 1RM significantly increased circulating BDNF in trained adult men, whereas lower-intensity protocols did not produce the same response. This result suggests that the BDNF response to RT may depend strongly on the clinical context. In people with MS, higher loads may impose greater physiological stress, central fatigue, and immunometabolic demand, potentially attenuating neurotrophic responsiveness [1,7]. Therefore, the optimal intensity for stimulating BDNF in MS may not be equivalent to that observed in healthy or trained individuals.

On the other hand, due to the limited number of studies, it is not feasible to specify an optimal exercise intensity. Therefore, we suggest caution when interpreting these findings; although some studies suggest a light-to-moderate intensity, given the limited number of experiments, any assertion remains merely speculative.

Disease phenotype and clinical status may also explain part of the variability in BDNF responsiveness. The positive studies included younger women with relapsing-remitting MS and mild-to-moderate disability, while Gravesteijn et al. [32] included individuals with progressive MS, older age, longer disease duration, and greater disability. In that study, participants had a mean age of approximately 54 years, median [IQR] EDSS of 4.5 [4.0–5.5], and disease duration of 20.2 ± 10.1 years. These characteristics may indicate a lower neuroplastic reserve and greater neurodegenerative burden. Thus, it is plausible that patients in earlier disease stages, particularly those with relapsing-remitting MS and lower EDSS scores, may present greater BDNF responsiveness to RT than individuals with progressive disease.

Another central methodological issue concerns the biological matrix used for BDNF quantification. The two studies reporting significant increases measured BDNF in serum [31,34], whereas the studies with non-significant findings measured BDNF in plasma collected with EDTA [32,33]. This distinction is critical because BDNF is largely stored in platelets and released during coagulation, which makes serum concentrations substantially higher than plasma concentrations [53]. Polacchini et al. [54] demonstrated that serum BDNF measurement is highly dependent on assay performance and pre-analytical procedures, including clotting time, centrifugation, storage, and kit variability. Therefore, serum and plasma BDNF should not be treated as interchangeable outcomes. This issue may partly explain the apparent discrepancy between positive and negative studies. Future trials should standardize BDNF collection protocols and report matrix-specific results separately, avoiding direct comparisons between serum and plasma estimates.

The present findings support RT as a promising adjunct strategy in MS rehabilitation. The pooled effect was positive and statistically significant, and all individual studies showed effect estimates in the same direction, ranging from SMD = 0.27 to 0.89. However, only one study demonstrated a statistically significant individual effect, and the overall certainty of evidence was low. Moreover, publication bias analyses should be interpreted with some caution because only four studies were included. Although Egger’s test (p = 0.115) and Begg and Mazumdar’s test (p = 0.083) did not indicate statistically significant asymmetry, the fail-safe N was only 9, and the trim-and-fill method suggested two potentially missing studies. These findings indicate that the pooled effect may be vulnerable to small-study effects and should be considered preliminary.

### Limitations

First, the number of included studies was small, limiting the stability of the pooled estimate and the reliability of publication bias analyses. Second, the included trials had small samples and methodological concerns related to randomization, allocation concealment, missing outcome data, and absence of prespecified biomarker analyses. Third, clinical heterogeneity was present across studies, including differences in MS phenotype, age, disease duration, EDSS, sex distribution, and training characteristics. Fourth, BDNF assessment was not standardized, particularly regarding serum versus plasma measurement. Finally, the GRADE assessment indicated low certainty of evidence, mainly due to serious risk of bias, imprecision, and methodological variability.

Future studies should prioritize adequately powered randomized controlled trials, prespecified BDNF outcomes, standardized blood collection procedures, and clear separation between serum and plasma BDNF. Trials should also compare different RT intensities and progression models, while stratifying participants by MS phenotype, EDSS, disease duration, sex, medication use, and fatigue status. In addition, future research should combine BDNF with neuroimaging outcomes, inflammatory markers, neurofilament light chain, functional tests, fatigue scales, and patient-reported outcomes to determine whether changes in peripheral BDNF are associated with meaningful clinical or neuroprotective adaptations.

### Strengths

First, this systematic review and meta-analysis was conducted in accordance with the recommendations of the Cochrane Handbook and the PRISMA guidelines, and it also has prospective registration in PROSPERO, increasing methodological transparency and reducing the risk of analytical bias. Second, exclusive inclusion of isolated RT interventions, which allowed for the specific investigation of the independent contribution of this modality to peripheral BDNF levels in individuals with MS, overcoming a frequent limitation of previous reviews that combined different types of exercise. Third, internationally recognized methodological tools were used to assess life expectancy and certainty of evidence, including RoB 2, ROBINS-I, and GRADE. Fourth, the statistical analysis also employed robust procedures for meta-analyses with a small number of studies, such as the random-effects model with REML estimator and Hartung-Knapp adjustment. Fifth, the absence of statistically relevant heterogeneity (I^2^ = 0%) and the directional consistency of effects across studies strengthen the reliability of the findings, even considering the specific limitations of the limited number of investigations found.

## Conclusion

Isolated RT may increase peripheral BDNF levels in individuals with MS, but this effect is supported by limited and low-certainty evidence. The current data suggest that BDNF responsiveness may be more evident after low-to-moderate intensity protocols and in less advanced clinical profiles, whereas high-intensity RT may produce benefits through BDNF-independent mechanisms. These findings reinforce the therapeutic potential of RT in MS, but also highlight the need for methodological standardization and more robust trials before firm conclusions can be drawn regarding its neurotrophic effects. Furthermore, given that only four studies were included in the meta-analysis, we suggest interpreting the results with some caution. We highlight that the literature still lacks experimental studies analyzing the isolated effect of strength training on BDNF levels in patients with multiple sclerosis.

## Supporting information

S1 File In Table S1, PRISMA checklist; In Table S2, presents the search strategy, with the respective access links and the database from which the articles were extracted; In Table S3, presents the List of excluded articles and their reasons. (DOCX)

S2 File Detailed systematic search spreadsheet. (XLSX)
