# Supplementary material for: Is resistance training effective to increase BDNF in multiple sclerosis? An updated systematic review and meta-analysis of isolated interventions
Source: PLoS One. 2026 Oct 27;21(10):e0359019. doi: 10.1371/journal.pone.0359019 (PMC13712535; doi:10.1371/journal.pone.0359019)
Supplement: Supplementary file 1 [file pone.0359019.s001.docx]

**Supplementary material**

**Table S1.** PRISMA Checklist

| **Item** | **PRISMA 2020 item** | **Location in manuscript** | **Comments** |
| --- | --- | --- | --- |
| **1** | Title | Title page | The title clearly identifies the article as a systematic review and meta-analysis. |
| **2** | Abstract | Abstract | The abstract reports background, objective, methods, results, and conclusion; PROSPERO registration number. |
| **3** | Rationale | Introduction | The rationale is clearly presented, emphasizing the uncertainty regarding the independent effect of isolated resistance training on BDNF in multiple sclerosis. |
| **4** | Objectives | End of Introduction | The objective is explicitly stated: to investigate the effects of isolated resistance training on peripheral BDNF levels in individuals with multiple sclerosis. |
| **5** | Eligibility criteria | Inclusion and Exclusion Criteria | Inclusion and exclusion criteria are described. Consider presenting them explicitly according to the PICOS framework. |
| **6** | Information sources | Search Strategy and Databases | Databases are reported: PubMed/MEDLINE, Web of Science, SciELO, Scopus, PEDro, and Cochrane Library. The final search date is reported as January 10, 2026. |
| **7** | Search strategy | Search Strategy and Databases; Supplementary Material S1 | The manuscript states that the complete search strategies are available in Supplementary Material S1. |
| **8** | Selection process | Study Selection and Data Extraction | The selection process was performed independently by two reviewers in two stages, with disagreements resolved by consensus or by a third reviewer. |
| **9** | Data collection process | Study Selection and Data Extraction | Data extraction was independently conducted by two reviewers using a standardized spreadsheet. |
| **10a** | Data items — outcomes | Study Selection and Data Extraction; Statistical Analysis | BDNF is clearly the main outcome. |
| **10b** | Data items — other variables | Study Selection and Data Extraction | The manuscript reports extraction of study characteristics, sample features, disease subtype, EDSS, intervention details, BDNF matrix, assay method, and quantitative data. |
| **11** | Study risk of bias assessment | Risk of Bias Assessment | RoB 2 was used for randomized trials, and ROBINS-I was used for the non-randomized controlled study. Independent assessment and consensus procedures are described. |
| **12** | Effect measures | Statistical Analysis | Effect sizes were expressed as standardized mean differences with 95% confidence intervals using Hedges’ correction. |
| **13a** | Synthesis methods — deciding eligibility for synthesis | Statistical Analysis | The manuscript describes the use of continuous data, including means, standard deviations, and sample sizes, for quantitative synthesis. |
| **13b** | Synthesis methods — data preparation | Statistical Analysis | The manuscript explains that effect sizes were calculated from pre- to post-intervention differences when necessary, with values estimated from figures when required. |
| **13c** | Synthesis methods — tabulation and visual display | Results; Table 1; Figures 1–3 | Study characteristics and results are presented in Table 1, and forest/funnel plots are provided. |
| **13d** | Synthesis methods — statistical model | Statistical Analysis | A random-effects model was used with REML estimation and Hartung–Knapp adjustment. |
| **13e** | Synthesis methods — heterogeneity and sensitivity | Statistical Analysis; Summary of Findings | Heterogeneity was assessed using Cochran’s Q, I², and τ². Leave-one-out sensitivity analysis was also performed. |
| **13f** | Synthesis methods — additional analyses | Statistical Analysis | Publication bias and sensitivity analyses are described. |
| **14** | Reporting bias assessment | Statistical Analysis; Publication Bias paragraph | Funnel plot, Egger’s regression, Begg and Mazumdar’s test, trim-and-fill, and fail-safe N were reported. |
| **15** | Certainty assessment | Certainty of Evidence; GRADE Table | The GRADE approach was applied, and the certainty of evidence was rated as low. |
| **16a** | Study selection — flow diagram | Results; Flowchart 1 | The manuscript reports the number of records identified, screened, excluded, assessed for eligibility, and included. |
| **16b** | Study selection — excluded studies with reasons | Results | The manuscript summarizes reasons for exclusion, but a supplementary table listing full-text excluded studies with specific reasons is recommended. |
| **17** | Study characteristics | Characteristics of the Studies; Table 1 | Characteristics of included studies are reported, including design, sample, intervention, BDNF assessment, and main findings. |
| **18** | Risk of bias in studies | Determining the Risk of Bias | Risk of bias judgments are described narratively for each included study. |
| **19** | Results of individual studies | Table 1; Summary of Findings | Individual study results and direction of effects are reported. |
| **20a** | Results of syntheses — contributing studies | Summary of Findings | The manuscript reports that four studies were included in the quantitative synthesis. |
| **20b** | Results of syntheses — pooled results and precision | Summary of Findings; Figure 1 | The Abstract/Results report SMD = 0.49; 95% CI: 0.15 to 0.83; Z = 2.80; p = 0.005**.** |
| **20c** | Results of syntheses — heterogeneity | Summary of Findings | Heterogeneity is reported as Q(3) = 1.681, p = 0.643, I² = 0%, and τ² = 0.000. |
| **20d** | Results of syntheses — sensitivity/additional analyses | Summary of Findings; Figure 2 | Leave-one-out analysis is reported, identifying Moghadasi et al. as influential/outlying. |
| **21** | Reporting biases | Publication Bias paragraph; Figure 3 | Reporting bias is assessed and interpreted cautiously due to the small number of studies. |
| **22** | Certainty of evidence | Analysis of the Strength of Evidence; Table 2 | The GRADE assessment is reported. |
| **23a** | Discussion — general interpretation | Discussion | The findings are interpreted in relation to the objective, previous literature, and clinical relevance. |
| **23b** | Discussion — limitations of included evidence | Limitations | The limitations of the included studies are clearly discussed, including small samples, risk of bias, heterogeneity, and BDNF measurement issues. |
| **23c** | Discussion — limitations of review process | Limitations | The review limitations are addressed. |
| **23d** | Discussion — implications | Discussion; Future Studies | Implications for rehabilitation and future research are well described. |
| **24a** | Registration and protocol | Methods | PROSPERO registration is reported: CRD420251141069. |
| **24b** | Access to protocol | Methods | The registration number is reported and the protocol is available in PROSPERO. |
| **24c** | Amendments to protocol | Not identified | A statement indicating whether there have been any changes to the protocol has been provided. |
| **25** | Support | Funding | Funding is reported as provided by the Goiás State Research Support Foundation — FAPEG. |
| **26** | Competing interests | Conflicts of Interest | The authors declare no conflicts of interest. |
| **27** | Availability of data, code, and other materials | Supplementary Material | As indicated in the Supplementary Material |

**Table S2.** Electronic complete search strategy across the different databases

**PubMed**

((((Multiple Sclerosis[Title]) AND (Resistance Training[Title])) OR (Strength Training[Title])) AND (BDNF[Title])) OR (brain derived neurotrophic factor[Title])

https://pubmed.ncbi.nlm.nih.gov/?term=%28%28%28%28Multiple+Sclerosis%5BTitle%5D%29+AND+%28Resistance+Training%5BTitle%5D%29%29+OR+%28Strength+Training%5BTitle%5D%29%29+AND+%28BDNF%5BTitle%5D%29%29+OR+%28brain+derived+neurotrophic+factor%5BTitle%5D%29&filter=pubt.clinicaltrial&filter=pubt.randomizedcontrolledtrial

**Web of Science (Clarivate)**

Multiple Sclerosis (Title) and Resistance Training (Title) or Strength Training (Title) and BDNF (Title) or brain derived neurotrophic factor (Title) and Randomized controlled trial (All Fields)

https://www.webofscience.com/wos/woscc/summary/2b12d1b4-e08c-4c4f-b484-7f7e818ea3cc-01a964c42d/relevance/1

**Scopus**

Multiple Sclerosis AND Resistance Training OR Strength Training AND BDNF OR brain derived neurotrophic factor AND Randomized Controlled trial AND ( LIMIT-TO ( DOCTYPE , "ar" ) ) AND ( LIMIT-TO ( EXACTKEYWORD , "Human" ) OR LIMIT-TO ( EXACTKEYWORD , "Controlled Study" ) OR LIMIT-TO ( EXACTKEYWORD , "Exercise" ) OR LIMIT-TO ( EXACTKEYWORD , "Humans" ) )

https://www.scopus.com/results/results.uri?sort=plf-f&src=s&sid=74bd1944da07a7b071a578ed105c5ef4&sot=a&sdt=cl&sl=141&s=Multiple+Sclerosis+AND+Resistance+Training+OR+Strength+Training+AND+BDNF+OR+brain+derived+neurotrophic+factor+AND+Randomized+Controlled+trial&origin=resultslist&editSaveSearch=&txGid=92638f495ecf56796d57684e719faeae&sessionSearchId=74bd1944da07a7b071a578ed105c5ef4&limit=10&cluster=scosubtype%2C%22ar%22%2Ct+scoexactkeywords%2C%22Human%22%2Ct%2C%22Controlled+Study%22%2Ct%2C%22Exercise%22%2Ct%2C%22Humans%22%2Ct

**Scielo**

(ti:(Multiple Sclerosis)) AND (ti:(Resistance Training)) OR (ti:(Strength Training)) AND (ti:(BDNF)) OR (ti:(brain derived neurotrophic factor ))

(Multiple Sclerosis) AND (Resistance Training) OR (Strength Training) AND (BDNF) OR (brain derived neurotrophic factor)

https://search.scielo.org/?q=%28Multiple%C2%A0Sclerosis%29+AND+%28Resistance%C2%A0Training%29+OR+%28Strength%C2%A0Training%29+AND+%28BDNF%29+OR+%28brain%C2%A0derived%C2%A0neurotrophic%C2%A0factor%29&lang=pt&filter%5Bin%5D%5B%5D=scl

**PEDro**

Multiple Sclerosis AND Resistance Training OR Strength Training AND "BDNF"

https://search.pedro.org.au/search-results?calc_text=multiple+sclerosis+and+resistance+training+and+%27%27BDNF%27%27&-find=Search

**Cochrane Data Base**

"multiple sclerosis" in Title Abstract Keyword AND "resistance training" in Title Abstract Keyword OR "strength training" in Title Abstract Keyword AND BDNF in Title Abstract Keyword AND "randomized controlled trial" in Title Abstract Keyword

**Table S3.** List of excluded articles and their reasons

| **Title** | **Source** | **Status** | **Reason** |
| --- | --- | --- | --- |
| (The effects of combined exercise training with aerobic dominant and coenzyme Q10 supplementation on serum BDNF and NGF levels in patients with multiple sclerosis) [Persian] | PEDRO | N | Combined Exercise |
| A brain-derived neurotrophic factor (BDNF) haplotype is associated with antidepressant treatment outcome in mood disorders | PUBMED | N | unrelated to population |
| Brain derived neurotrophic factor in multiple sclerosis: effect of 24 weeks endurance and resistance training | PUBMED | N | Combined exercise |
| Brain-Derived Neurotrophic Factor in Multiple Sclerosis Disability: A Prospective Study | Scopus | N | unrelated to outcome |
| Brain-derived neurotrophic factor levels under chronic natalizumab treatment in multiple sclerosis. A preliminary report | PUBMED | N | unrelated to outcome |
| Decrease of urinary nerve growth factor but not brain-derived neurotrophic factor in patients with interstitial cystitis/bladder pain syndrome treated with hyaluronic acid | PUBMED | N | unrelated to outcome |
| Effect of combined exercise training on serum brain-derived neurotrophic factor, suppressors of cytokine signaling 1 and 3 in patients with multiple sclerosis | PUBMED | N | Combined exercise |
| Effect of glatiramer acetate on peripheral blood brain-derived neurotrophic factor and phosphorylated TrkB levels in relapsing-remitting multiple sclerosis | PUBMED | N | unrelated to outcome |
| Effects of concurrent training and CoQ10 on neurotrophic factors and physical function in people with Multiple Sclerosis: a pilot study | Scopus | N | unrelated to outcome |
| Effects of long-term administration of Multi-Strain Probiotic on circulating levels of BDNF, NGF, IL-6 and mental health in patients with multiple sclerosis: a randomized, double-blind, placebo-controlled trial | Scopus | N | unrelated to outcome |
| Evolution of brain-derived neurotrophic factor levels after autologous hematopietic stem cell transplantation in multiple sclerosis | PUBMED | N | unrelated to outcome |
| Exercise improves neurotrophins in multiple sclerosis independent of disability status | Scopus | N | Combined Exercise |
| Exercise Improves Physical Capacity, Cognition, Quality of Life and Promotes Neurotrophic Factors in Patients with Multiple Sclerosis | Scopus | N | Aerobic exercise |
| Exercise-induced changes in neurotrophic factors and markers of blood-brain barrier permeability are moderated by weight status in multiple sclerosis | Scopus | N | Aerobic exercise |
| Immediate and short-term effect of physical exercise on BDNF in multiple sclerosis patients: a systematic review and meta-analysis | Scopus | N | Combined Exercise |
| Impact of cognitive-aerobic exercise training on brain-derived neurotrophic factor, dual-tasking abilities, and mood state in individuals with multiple sclerosis | PUBMED | N | Aerobic exercise |
| Lower brain-derived neurotrophic factor in serum of relapsing remitting MS: reversal by glatiramer acetate | PUBMED | N | unrelated to outcome |
| Production of brain-derived neurotrophic factor by mononuclear cells of patients with multiple sclerosis treated with glatiramer acetate, interferon-beta 1a, and high doses of immunoglobulins | PUBMED | N | Unrelated outcome |
| Serum brain-derived neurotrophic factor response to aerobic exercise in multiple sclerosis | PUBMED | N | Aerobic exercise |
| The effect of 12-weeks resistance and endurance training on the serum levels NGF, BDNF, and VDBP in women with multiple sclerosis | Web of Science | N | Combined Exercise |
| The effect of combined functional training on BDNF, IGF-1, and their association with health-related fitness in the multiple sclerosis women | Scopus | N | Combined Exercise |
| The effect of interferon-β therapy on brain-derived neurotrophic factor serum concentration in relapsing-remitting multiple sclerosis: a randomized clinical trial | Web of Science | N | intervention differrent |
