# Supplementary material for: Is resistance training effective to increase BDNF in multiple sclerosis? An updated systematic review and meta-analysis of isolated interventions
Source: PLoS One. 2026 Oct 27;21(10):e0359019. doi: 10.1371/journal.pone.0359019 (PMC13712535; doi:10.1371/journal.pone.0359019)
Supplement: Supplementary file 2 [file pone.0359019.s004.docx]

**Marcelo Magalhães Sales, PhD.**

**Graduate Program in Environment and Society at the State University of Goiás**

**Brazil. Avenida Brasil, número 435, Conjunto Setor Hélio Leão, Quirinópolis - GO, 75862-196**

**Telefone: +55 (64) 3651-2285**

**E-mail: marcelomagalhaessales@gmail.com**

July 7^th^, 2026

To

***Emily Chenette, PhD***

***Editor-in-Chief***

***PLOS One***

Ref. Revised version of the manuscript: “Is Resistance Training Effective to Increase BDNF in Multiple Sclerosis? An Updated Systematic Review and Meta-Analysis of Isolated Interventions”.

Dear Editor,

First of all, we appreciate the comments about our manuscript. We considered all comments and now we would like to resubmit this manuscript to the **PLOS One** highlighting the revisions carried out since the original submission and providing responses point-by-point to the comments. All modifications are highlighted in yellow.

**EDITOR´S COMMENTS**

**Comment 1. Please ensure that your manuscript meets PLOS ONE's style requirements, including those for file naming. The PLOS ONE style templates can be found at**

[**https://journals.plos.org/plosone/s/file?id=wjVg/PLOSOne_formatting_sample_main_body.pdf**](https://journals.plos.org/plosone/s/file?id=wjVg/PLOSOne_formatting_sample_main_body.pdf) **and**

[**https://journals.plos.org/plosone/s/file?id=ba62/PLOSOne_formatting_sample_title_authors_affiliations.pdf**](https://journals.plos.org/plosone/s/file?id=ba62/PLOSOne_formatting_sample_title_authors_affiliations.pdf)

**REPLY:** Thank you. The manuscript has been extensively revised in accordance with the reviewers' recommendations and the journal's guidelines.

**Comment 2. We note that your Data Availability Statement is currently as follows: [All the data for this article can be found in the body of the text or in supplementary material]. Please confirm at this time whether or not your submission contains all raw data required to replicate the results of your study. Authors must share the “minimal data set” for their submission. PLOS defines the minimal data set to consist of the data required to replicate all study findings reported in the article, as well as related metadata and methods (https://journals.plos.org/plosone/s/data-availability#loc-minimal-data-set-definition).**

**For example, authors should submit the following data:**

**- The values behind the means, standard deviations and other measures reported;**

**- The values used to build graphs;**

**- The points extracted from images for analysis.**

**Authors do not need to submit their entire data set if only a portion of the data was used in the reported study.**

**If your submission does not contain these data, please either upload them as Supporting Information files or deposit them to a stable, public repository and provide us with the relevant URLs, DOIs, or accession numbers. For a list of recommended repositories, please see** [**https://journals.plos.org/plosone/s/recommended-repositories**](https://journals.plos.org/plosone/s/recommended-repositories)**.**

**REPLY:** All data comprising this manuscript have been made available, as recommended by the editorial team. This is a meta-analysis; therefore, the manuscript presents all the necessary data. The supplementary material complements the data.

**Comment 3. If there are ethical or legal restrictions on sharing a de-identified data set, please explain them in detail (e.g., data contain potentially sensitive information, data are owned by a third-party organization, etc.) and who has imposed them (e.g., an ethics committee). Please also provide contact information for a data access committee, ethics committee, or other institutional body to which data requests may be sent. If data are owned by a third party, please indicate how others may request data access.**

**REPLY:** As this is a systematic review with meta-analysis, approval from an ethics committee is not required. However, as explicitly stated in the manuscript, the protocol adopted for this study was duly registered on the PROSPERO platform under the number CRD420251141069. We appreciate the opportunity to provide this clarification.

**Comment 4. Please include captions for your Supporting Information files at the end of your manuscript, and update any in-text citations to match accordingly. Please see our Supporting Information guidelines for more information:** [**http://journals.plos.org/plosone/s/supporting-information**](http://journals.plos.org/plosone/s/supporting-information)**.**

**REPLY:** We thank you again for the support. Our manuscript contains only one supplementary material table, which we have designated Table S1. We have included a caption for this table as requested by the editorial team. This addition is highlighted in yellow in the manuscript. Please see below:

**Caption:**

**Table S1.** Supplementary material presents the search strategy, with the respective access links and the database from which the articles were extracted.

We are going to submit it to the journal. However, if you are unable to view it properly, please check the link below.

<https://docs.google.com/document/d/17zR-wj8hxt2GtWAr_E-zzpj3ZvllBwqs/edit?usp=sharing&ouid=111741925622305591348&rtpof=true&sd=true>

**Comment 5. If the reviewer comments include a recommendation to cite specific previously published works, please review and evaluate these publications to determine whether they are relevant and should be cited. There is no requirement to cite these works unless the editor has indicated otherwise.**

**REPLY:** We appreciate the editorial team's diligence. On the other hand, it is worth noting that no request of this nature was made by any of the reviewers.

**REVIEWER #1**

**This study aims to conduct a systematic review and meta-analysis of the effects of isolated resistance training (RT) on peripheral BDNF levels in individuals with multiple sclerosis (MS). The methods are clearly described, and the interpretation of the findings is appropriate. I have only a few minor comments for the authors to consider:**

**Comment 1. In the Abstract, line 73, what does “≈80% 1RM” mean? Please also define RM at first use.**

**REPLY:** Although the acronym "1 RM" seemed universally known to those familiar with this type of literature, the question raised by the esteemed reviewer made it evident that this is not the case. Therefore, we have included the full term before introducing the acronym. We thank you for helping us improve the manuscript. For clarification, "1 RM" stands for "one-repetition maximum"; it refers to the load a subject is capable of lifting a single time. We have inserted the sentence below exactly where the esteemed reviewer indicated. From that point onward, we use the previously mentioned acronym "1RM." This insertion is highlighted in yellow in the manuscript. Please see below:

[...] (≈80% of one-repetition maximum: 1RM) [...].

**Comment 2. In the Introduction, lines 103–104, if specific recommendations exist regarding the types of exercise used in MS rehabilitation, it would be helpful to describe them explicitly. For example, do the guidelines recommend RT alone or a combination of RT and aerobic exercise?**

**REPLY:** We appreciate the opportunity to clarify this point. Both aerobic and resistance exercises are recommended. Therefore, as suggested, we have added a sentence highlighting this aspect and the position statement recommending both aerobic and resistance exercises. This addition is highlighted in yellow in the manuscript. Please see below:

[...] It is worth noting that both aerobic and resistance exercises are recommended by the position statement on exercise for people with mild to moderate multiple sclerosis from Exercise & Sports Science Australia (ESSA) (Hoang et al., 2022). [...]

**Comment 3.** **In lines 306–315, please provide additional information about the control conditions in the included RCTs. Did participants in the control groups receive no intervention or any specific instructions? This information would complement the description of the interventions.**

**REPLY:** We thank you again for the opportunity to clarify this point. In none of the studies deemed eligible for inclusion in this review and meta-analysis did the individuals in the control group receive any type of intervention. As requested, we have inserted the sentence below to report this information. We would like to emphasize that all changes are highlighted in yellow in the manuscript.

[…] It is worth noting that, in all studies eligible for inclusion in the present study, individuals in the control group did not receive any type of intervention. […]

**Comment 4.** **In Figures 1 and 2, the first group is labelled “BDNF,” whereas this appears to represent the RT intervention group. BDNF is the outcome rather than the intervention, right? Please revise.**

**REPLY:** The reviewer is entirely correct. We have made the change as requested. Please forgive us for this oversight.

**Comment 5.** **Again, in Figures 1 and 2, the labels below the x-axis, “Grupo Controle” and “Favor BNDF”, does not appear accurate. Please revise them to, for example, “Favours control” and “Favours RT.”**

**REPLY:** The reviewer is entirely correct. We have made the change as requested. Please forgive us for this oversight.

**REVIEWER #2**

**This manuscript addresses a relevant and well-defined research question regarding the effects of isolated resistance training on peripheral BDNF levels in individuals with multiple sclerosis. The topic is appropriate for a systematic review and meta-analysis, and the authors should be commended for registering the review protocol, following established systematic review methodologies, and applying recognized tools for risk-of-bias and certainty-of-evidence assessment. Overall, I believe the manuscript has the potential to make a useful contribution to the literature. However, several issues should be addressed before the work can be considered for publication.**

**Major Comments**

**Comment 1.** **Statistical reporting requires clarification and harmonization. The manuscript reports inconsistent pooled effect estimates across different sections. Confidence intervals, test statistics, and p-values associated with the primary meta-analysis should be verified and reported consistently throughout the abstract, results, discussion, tables, and figures. In addition, the manuscript states that a Hartung-Knapp adjustment was used, yet significance testing appears to be reported using Z statistics. The authors should clearly describe the inferential framework used and ensure that all reported results correspond to the final analytical model.**

**REPLY:** Please accept our apologies. The highlighted sections of the text (abstract, statistical analysis, results, and discussion) have undergone extensive revision, and we believe we have corrected the mentioned inconsistencies. Regarding the Hartung–Knapp adjustment, we have inserted the values as requested. Please refer to the excerpts below, which we have added and highlighted in yellow in the manuscript.

**Abstract:**

[…] With the Hartung–Knapp adjustment, due to the reduced number of studies, the analysis continued to show a positive intervention effect: (SMD=0.49; 95%CI: 0.42 to 0.56; t=3.716; p=0.0339). […]

**Statistical Analysis**

**[…]** together with the Hartung–Knapp adjustment (*Student’s t* – distribution), which is considered more robust for meta-analyses including a small number of studies. The Hartung–Knapp adjustment was applied to account for statistical uncertainty, particularly when few studies are involved (Röver et al., 2015). This adjustment involves modifying the reference distribution by replacing the critical value (*Z*_1−α/2_) with the critical value (*t*_1−α/2; k−1_) and setting the degrees of freedom to (*k*−1), where *k* is the total number of studies included in the meta-analysis (IntHout et al., 2014). Additionally, the standard error is adjusted by multiplying the standard standard error by a correction factor (*q*), calculated based on Cochrane’s *Q* statistic and the study weights. […]

[…] The statistical significance of the overall effect was determined using the Z test. In the adjusted analysis, the critical Z-value was replaced by the critical value of the Student's t-distribution, and the degrees of freedom were defined as (k−1), where k is the total number of studies included in the meta-analysis (IntHout et al., 2014). The significance level was set at 5% (p < 0.05). The direction of effects was standardized so that positive SMD values indicated a benefit of the intervention compared to the control group. […]

**Results**

[…] In addition, with the Hartung–Knapp adjustment, due to the reduced number of studies, the analysis continued to show a positive intervention effect: (SMD=0.49; 95%CI: 0.42 to 0.56; t=3.716; p=0.0339).[…]

**Comment 2.** **Limited evidence base and imprecision. The meta-analysis includes only four studies with a relatively small total sample size. Although the pooled effect suggests a beneficial influence of resistance training on BDNF, the evidence remains limited and imprecise. The conclusions should reflect this uncertainty more explicitly.**

**REPLY:** We agree with the esteemed reviewer. We have rephrased the conclusion and highlighted it in yellow within the manuscript; we believe the current wording addresses the reviewer's request. Please see below:

[…] Furthermore, given that only four studies were included in the meta-analysis, we suggest interpreting the results with some caution. We highlight that the literature still lacks experimental studies analyzing the isolated effect of strength training on BDNF levels in patients with multiple sclerosis.[…]

**Comment 3.** **Clinical and methodological heterogeneity. Included studies differ with respect to disease phenotype, disability status, intervention characteristics, intervention duration, training intensity, and methods used to measure BDNF (serum versus plasma). These differences should be discussed more thoroughly as potential sources of heterogeneity and limitations to interpretation.**

**REPLY:** We appreciate the reviewer's comment; however, we would like to emphasize that, although the studies exhibit the mentioned differences, these were not sufficient to alter the conclusions of the present study. In other words, regardless of these differences, the results indicate that isolated resistance training appears effective in inducing some increase in BDNF levels, even after adjusted analysis. Furthermore, the heterogeneity analysis did not reveal significant variability among the studies. Cochran’s Q test indicated non-significant heterogeneity (Q(3) = 1.681; p = 0.643), and the I² statistic was 0%, with a between-study variance estimate (τ²) of 0.000. These findings suggest that the observed variability in effect sizes is likely attributable to sampling error rather than actual differences between studies, indicating a high degree of consistency in the included evidence. Furthermore, to some extent, it appears we were able to address certain aspects raised by the esteemed reviewer. Please refer to lines 527–579. In that section, we discuss exercise intensity, disease phenotype, clinical status, and the biological matrix used for quantification. Thus, we believe that the manuscript, in its current form, adequately addresses the interpretation of the findings and explicitly highlights the limitations as well as the relative strength of our results.

**Comment 4.** **Data transparency and reproducibility. The Data Availability Statement indicates that data are available within the manuscript and supplementary materials. However, the extraction dataset used to generate the meta-analysis is not clearly available. Providing the complete extraction spreadsheet, calculated effect sizes, and any transformed or estimated values would improve transparency and reproducibility.**

**REPLY:** Please forgive us. There was a mistake on our part. The supplementary material has been made available as requested.

**Comment 5.** **Interpretation of training intensity effects. The suggestion that low- or moderate-intensity resistance training may be more effective than higher-intensity protocols appears to be based on indirect comparisons across a very small number of studies. Similarly, statements regarding potential BDNF-independent mechanisms underlying benefits from higher-intensity training are speculative and should be toned down unless directly supported by the available evidence.**

**REPLY:** We have inserted the sentence below to address the reviewer's request to some extent. It is worth noting that this addition is highlighted in yellow in the manuscript.

[…] On the other hand, due to the limited number of studies, it is not feasible to specify an optimal exercise intensity. Therefore, we suggest caution when interpreting these findings; although some studies suggest a light-to-moderate intensity, given the limited number of experiments, any assertion remains merely speculative. […]

**Minor Comments**

**Comment 6.** **The manuscript is generally understandable; however, the English language requires revision. Several grammatical errors, typographical mistakes, and awkward expressions are present throughout the text. Professional language editing is recommended.**

**REPLY:** We have carefully revised the entire manuscript to improve the English language, grammar, punctuation, typographical accuracy, and overall readability. Awkward expressions were rewritten to improve clarity and scientific flow, and several minor errors were corrected throughout the text. We believe that the revised version is now clearer and more suitable for publication.

**Comment 7.** **Some terminology and formatting inconsistencies should be corrected to improve clarity and readability.**

**REPLY:** We thank you again for helping to improve the manuscript. We would like to highlight that the manuscript has undergone extensive revision.

**Comment 8.** **The authors should ensure that all supplementary materials, tables, and figure references are correctly labeled and consistently cited in the text.**

**REPLY:** Yes!!

**Comment 9. Additional detail regarding data extraction procedures, handling of missing data, and any statistical conversions would strengthen methodological transparency.**

**REPLY:** Thank you. As previously mentioned, we have made a supplementary material table available.

**Comment 10. Overall Assessment. The review addresses an important clinical and biological question and applies generally appropriate systematic review and meta-analytic methods. The primary conclusion—that isolated resistance training may increase peripheral BDNF levels in individuals with multiple sclerosis—is broadly supported by the available evidence. However, limitations related to the small evidence base, statistical reporting inconsistencies, data transparency, and interpretation of findings require substantial revision.**

**REPLY:** Thank you for helping us significantly improve the manuscript.
