# Supplementary material for: Is resistance training effective to increase BDNF in multiple sclerosis? An updated systematic review and meta-analysis of isolated interventions
Source: PLoS One. 2026 Oct 27;21(10):e0359019. doi: 10.1371/journal.pone.0359019 (PMC13712535; doi:10.1371/journal.pone.0359019)
Supplement: Supplementary file 3 [file pone.0359019.s005.docx]

PONE-D-26-24021R1
Is Resistance Training Effective to Increase BDNF in Multiple Sclerosis? An Updated Systematic Review and Meta-Analysis of Isolated Interventions

PLOS One

**Journal Requirements:**

**Comment 1.** If the reviewer comments include a recommendation to cite specific previously published works, please review and evaluate these publications to determine whether they are relevant and should be cited. There is no requirement to cite these works unless the editor has indicated otherwise.

**REPLY:** OK, understood. The reviewers did not require the inclusion of any out-of-scope references.

**Comment 2.** Please review your reference list to ensure that it is complete and correct. If you have cited papers that have been retracted, please include the rationale for doing so in the manuscript text, or remove these references and replace them with relevant current references. Any changes to the reference list should be mentioned in the rebuttal letter that accompanies your revised manuscript. If you need to cite a retracted article, indicate the article’s retracted status in the References list and also include a citation and full reference for the retraction notice.

**REPLY:** All references were included using EndNote software. All are relevant to the study. No references have been retracted.

Reviewer's Responses to Questions

**Comments to the Author**

Reviewer #1: In Figure 1 and Figure 2, the group heading is still BNDF and CONTROL. I understand the mean and SD values shown are the BNDF values. The group heading should be something like Intervention group and Control group, rather than BNDF. Please correct.

**REPLY:** We apologize for the error. The reviewer is indeed correct. We had previously adjusted the figure but overlooked other important elements. Consider it done. Observe the adjusted figures below.

**Figure 1.** Forest plot of the effects of the intervention on BDNF levels.

**Figure 2.** Forest plot from sensitivity analysis on BDNF levels.
