# Supplementary material for: Is resistance training effective to increase BDNF in multiple sclerosis? An updated systematic review and meta-analysis of isolated interventions
Source: PLoS One. 2026 Oct 27;21(10):e0359019. doi: 10.1371/journal.pone.0359019 (PMC13712535; doi:10.1371/journal.pone.0359019)
Supplement: Peer review history

# Peer review: Decision Letter 0

**DOI:** 10.1371/journal.pone.0359019.r001

**Authors:** Tanja Grubić Kezele

22 Jun 2026

PONE-D-26-24021

Is Resistance Training Effective to Increase BDNF in Multiple Sclerosis? An Updated Systematic Review and Meta-Analysis of Isolated Interventions

PLOS One?

Dear Dr. Cameia,

Thank you for submitting your manuscript to PLOS ONE. After careful consideration, we feel that it has merit but does not fully meet PLOS ONE’s publication criteria as it currently stands. Therefore, we invite you to submit a revised version of the manuscript that addresses the points raised during the review process.

Based on the reviewers' suggestions, the paper needs a major revision. The reviewers' comments can be found below.

Please submit your revised manuscript by Aug 21 2026 11:59PM. If you will need more time than this to complete your revisions, please reply to this message or contact the journal office at plosone@plos.org. When you're ready to submit your revision, log on to https://www.editorialmanager.com/pone/ and select the 'Submissions Needing Revision' folder to locate your manuscript file.

- A letter that responds to each point raised by the academic editor and reviewer(s). You should upload this letter as a separate file labeled 'Response to Reviewers'.
- A marked-up copy of your manuscript that highlights changes made to the original version. You should upload this as a separate file labeled 'Revised Manuscript with Track Changes'.
- An unmarked version of your revised paper without tracked changes. You should upload this as a separate file labeled 'Manuscript'.

If you would like to make changes to your financial disclosure, please include your updated statement in your cover letter. Guidelines for resubmitting your figure files are available below the reviewer comments at the end of this letter.

If applicable, we recommend that you deposit your laboratory protocols in protocols.io to enhance the reproducibility of your results. Protocols.io assigns your protocol its own identifier (DOI) so that it can be cited independently in the future. For instructions see: https://journals.plos.org/plosone/s/submission-guidelines#loc-laboratory-protocols. Additionally, PLOS ONE offers an option for publishing peer-reviewed Lab Protocol articles, which describe protocols hosted on protocols.io. Read more information on sharing protocols at https://plos.org/protocols?utm_medium=editorial-email&utm_source=authorletters&utm_campaign=protocols.

As the corresponding author, your ORCID iD is verified in the submission system and will appear in the published article. PLOS supports the use of ORCID, and we encourage all coauthors to register for an ORCID iD and use it as well. Please encourage your coauthors to verify their ORCID iD within the submission system before final acceptance, as unverified ORCID iDs will not appear in the published article. *Only* the individual author can complete the verification step; PLOS staff *cannot* verify ORCID iDs on behalf of authors.

We look forward to receiving your revised manuscript.

Kind regards,

Tanja Grubić Kezele, Ph.D., M.D.

Academic Editor

PLOS One

Journal Requirements:

When submitting your revision, we need you to address these additional requirements.

1. Please ensure that your manuscript meets PLOS ONE's style requirements, including those for file naming. The PLOS ONE style templates can be found at

https://journals.plos.org/plosone/s/file?id=wjVg/PLOSOne_formatting_sample_main_body.pdf and

https://journals.plos.org/plosone/s/file?id=ba62/PLOSOne_formatting_sample_title_authors_affiliations.pdf

2. We note that your Data Availability Statement is currently as follows: [All the data for this article can be found in the body of the text or in supplementary material.]

Please confirm at this time whether or not your submission contains all raw data required to replicate the results of your study. Authors must share the “minimal data set” for their submission. PLOS defines the minimal data set to consist of the data required to replicate all study findings reported in the article, as well as related metadata and methods (https://journals.plos.org/plosone/s/data-availability#loc-minimal-data-set-definition).

For example, authors should submit the following data:

- The values behind the means, standard deviations and other measures reported;

- The values used to build graphs;

- The points extracted from images for analysis.

Authors do not need to submit their entire data set if only a portion of the data was used in the reported study.

If your submission does not contain these data, please either upload them as Supporting Information files or deposit them to a stable, public repository and provide us with the relevant URLs, DOIs, or accession numbers. For a list of recommended repositories, please see https://journals.plos.org/plosone/s/recommended-repositories.

If there are ethical or legal restrictions on sharing a de-identified data set, please explain them in detail (e.g., data contain potentially sensitive information, data are owned by a third-party organization, etc.) and who has imposed them (e.g., an ethics committee). Please also provide contact information for a data access committee, ethics committee, or other institutional body to which data requests may be sent. If data are owned by a third party, please indicate how others may request data access.

3. Please include captions for your Supporting Information files at the end of your manuscript, and update any in-text citations to match accordingly. Please see our Supporting Information guidelines for more information: http://journals.plos.org/plosone/s/supporting-information.

4. If the reviewer comments include a recommendation to cite specific previously published works, please review and evaluate these publications to determine whether they are relevant and should be cited. There is no requirement to cite these works unless the editor has indicated otherwise.

[Note: HTML markup is below. Please do not edit.]

Reviewers' comments:

Reviewer's Responses to Questions

**Comments to the Author**

1. Is the manuscript technically sound, and do the data support the conclusions?

Reviewer #1: Yes

Reviewer #2: Partly

**********

2. Has the statistical analysis been performed appropriately and rigorously?

Reviewer #1: Yes

Reviewer #2: No

**********

3. Have the authors made all data underlying the findings in their manuscript fully available?

The PLOS Data policy

Reviewer #1: Yes

Reviewer #2: No

**********

4. Is the manuscript presented in an intelligible fashion and written in standard English?

Reviewer #1: Yes

Reviewer #2: Yes

**********

Reviewer #1: This study aims to conduct a systematic review and meta-analysis of the effects of isolated resistance training (RT) on peripheral BDNF levels in individuals with multiple sclerosis (MS). The methods are clearly described, and the interpretation of the findings is appropriate. I have only a few minor comments for the authors to consider:

1. In the Abstract, line 73, what does “≈80% 1RM” mean? Please also define RM at first use.

2. In the Introduction, lines 103–104, if specific recommendations exist regarding the types of exercise used in MS rehabilitation, it would be helpful to describe them explicitly. For example, do the guidelines recommend RT alone or a combination of RT and aerobic exercise?

3. In lines 306–315, please provide additional information about the control conditions in the included RCTs. Did participants in the control groups receive no intervention or any specific instructions? This information would complement the description of the interventions.

4. In Figures 1 and 2, the first group is labelled “BDNF,” whereas this appears to represent the RT intervention group. BDNF is the outcome rather than the intervention, right? Please revise.

5. Again, in Figures 1 and 2, the labels below the x-axis, “Grupo Controle” and “Favor BNDF”, does not appear accurate. Please revise them to, for example, “Favours control” and “Favours RT.”

Reviewer #2: Reviewer Comments to the Authors

This manuscript addresses a relevant and well-defined research question regarding the effects of isolated resistance training on peripheral BDNF levels in individuals with multiple sclerosis. The topic is appropriate for a systematic review and meta-analysis, and the authors should be commended for registering the review protocol, following established systematic review methodologies, and applying recognized tools for risk-of-bias and certainty-of-evidence assessment.

Overall, I believe the manuscript has the potential to make a useful contribution to the literature. However, several issues should be addressed before the work can be considered for publication.

Major Comments

Statistical reporting requires clarification and harmonization.

The manuscript reports inconsistent pooled effect estimates across different sections. Confidence intervals, test statistics, and p-values associated with the primary meta-analysis should be verified and reported consistently throughout the abstract, results, discussion, tables, and figures. In addition, the manuscript states that a Hartung-Knapp adjustment was used, yet significance testing appears to be reported using Z statistics. The authors should clearly describe the inferential framework used and ensure that all reported results correspond to the final analytical model.

Limited evidence base and imprecision.

The meta-analysis includes only four studies with a relatively small total sample size. Although the pooled effect suggests a beneficial influence of resistance training on BDNF, the evidence remains limited and imprecise. The conclusions should reflect this uncertainty more explicitly.

Clinical and methodological heterogeneity.

Included studies differ with respect to disease phenotype, disability status, intervention characteristics, intervention duration, training intensity, and methods used to measure BDNF (serum versus plasma). These differences should be discussed more thoroughly as potential sources of heterogeneity and limitations to interpretation.

Data transparency and reproducibility.

The Data Availability Statement indicates that data are available within the manuscript and supplementary materials. However, the extraction dataset used to generate the meta-analysis is not clearly available. Providing the complete extraction spreadsheet, calculated effect sizes, and any transformed or estimated values would improve transparency and reproducibility.

Interpretation of training intensity effects.

The suggestion that low- or moderate-intensity resistance training may be more effective than higher-intensity protocols appears to be based on indirect comparisons across a very small number of studies. Similarly, statements regarding potential BDNF-independent mechanisms underlying benefits from higher-intensity training are speculative and should be toned down unless directly supported by the available evidence.

Minor Comments

The manuscript is generally understandable; however, the English language requires revision. Several grammatical errors, typographical mistakes, and awkward expressions are present throughout the text. Professional language editing is recommended.

Some terminology and formatting inconsistencies should be corrected to improve clarity and readability.

The authors should ensure that all supplementary materials, tables, and figure references are correctly labeled and consistently cited in the text.

Additional detail regarding data extraction procedures, handling of missing data, and any statistical conversions would strengthen methodological transparency.

Overall Assessment

The review addresses an important clinical and biological question and applies generally appropriate systematic review and meta-analytic methods. The primary conclusion—that isolated resistance training may increase peripheral BDNF levels in individuals with multiple sclerosis—is broadly supported by the available evidence. However, limitations related to the small evidence base, statistical reporting inconsistencies, data transparency, and interpretation of findings require substantial revision.

Recommendation: Major Revision.

**********

what does this mean?). If published, this will include your full peer review and any attached files.

If you choose “no”, your identity will remain anonymous but your review may still be made public.

**Do you want your identity to be public for this peer review?** For information about this choice, including consent withdrawal, please see our Privacy Policy

Reviewer #1: No

Reviewer #2: **Yes:**  I confirm that this review is my own work and is not submitted on behalf of another person

**********

[NOTE: If reviewer comments were submitted as an attachment file, they will be attached to this email and accessible via the submission site. Please log into your account, locate the manuscript record, and check for the action link "View Attachments". If this link does not appear, there are no attachment files.]

To ensure your figures meet our technical requirements, please review our figure guidelines: https://journals.plos.org/plosone/s/figures

You may also use PLOS’s free figure tool, NAAS, to help you prepare publication quality figures: https://journals.plos.org/plosone/s/figures#loc-tools-for-figure-preparation.

NAAS will assess whether your figures meet our technical requirements by comparing each figure against our figure specifications.

# Author comment: Author response to Decision Letter 1

**DOI:** 10.1371/journal.pone.0359019.r002

5 Aug 2026

Dear Editor,

First of all, we appreciate the comments about our manuscript. We considered all comments and now we would like to resubmit this manuscript to the PLOS One highlighting the revisions carried out since the original submission and providing responses point-by-point to the comments. All modifications are highlighted in yellow.

EDITOR´S COMMENTS

Comment 1. Please ensure that your manuscript meets PLOS ONE's style requirements, including those for file naming. The PLOS ONE style templates can be found at

https://journals.plos.org/plosone/s/file?id=wjVg/PLOSOne_formatting_sample_main_body.pdf and

https://journals.plos.org/plosone/s/file?id=ba62/PLOSOne_formatting_sample_title_authors_affiliations.pdf

REPLY: Thank you. The manuscript has been extensively revised in accordance with the reviewers' recommendations and the journal's guidelines.

Comment 2. We note that your Data Availability Statement is currently as follows: [All the data for this article can be found in the body of the text or in supplementary material]. Please confirm at this time whether or not your submission contains all raw data required to replicate the results of your study. Authors must share the “minimal data set” for their submission. PLOS defines the minimal data set to consist of the data required to replicate all study findings reported in the article, as well as related metadata and methods (https://journals.plos.org/plosone/s/data-availability#loc-minimal-data-set-definition).

For example, authors should submit the following data:

- The values behind the means, standard deviations and other measures reported;

- The values used to build graphs;

- The points extracted from images for analysis.

Authors do not need to submit their entire data set if only a portion of the data was used in the reported study.

If your submission does not contain these data, please either upload them as Supporting Information files or deposit them to a stable, public repository and provide us with the relevant URLs, DOIs, or accession numbers. For a list of recommended repositories, please see https://journals.plos.org/plosone/s/recommended-repositories.

REPLY: All data comprising this manuscript have been made available, as recommended by the editorial team. This is a meta-analysis; therefore, the manuscript presents all the necessary data. The supplementary material complements the data.

Comment 3. If there are ethical or legal restrictions on sharing a de-identified data set, please explain them in detail (e.g., data contain potentially sensitive information, data are owned by a third-party organization, etc.) and who has imposed them (e.g., an ethics committee). Please also provide contact information for a data access committee, ethics committee, or other institutional body to which data requests may be sent. If data are owned by a third party, please indicate how others may request data access.

REPLY: As this is a systematic review with meta-analysis, approval from an ethics committee is not required. However, as explicitly stated in the manuscript, the protocol adopted for this study was duly registered on the PROSPERO platform under the number CRD420251141069. We appreciate the opportunity to provide this clarification.

Comment 4. Please include captions for your Supporting Information files at the end of your manuscript, and update any in-text citations to match accordingly. Please see our Supporting Information guidelines for more information: http://journals.plos.org/plosone/s/supporting-information.

REPLY: We thank you again for the support. Our manuscript contains only one supplementary material table, which we have designated Table S1. We have included a caption for this table as requested by the editorial team. This addition is highlighted in yellow in the manuscript. Please see below:

Caption:

Table S1. Supplementary material presents the search strategy, with the respective access links and the database from which the articles were extracted.

We are going to submit it to the journal. However, if you are unable to view it properly, please check the link below.

https://docs.google.com/document/d/17zR-wj8hxt2GtWAr_E-zzpj3ZvllBwqs/edit?usp=sharing&ouid=111741925622305591348&rtpof=true&sd=true

Comment 5. If the reviewer comments include a recommendation to cite specific previously published works, please review and evaluate these publications to determine whether they are relevant and should be cited. There is no requirement to cite these works unless the editor has indicated otherwise.

REPLY: We appreciate the editorial team's diligence. On the other hand, it is worth noting that no request of this nature was made by any of the reviewers.

REVIEWER #1

This study aims to conduct a systematic review and meta-analysis of the effects of isolated resistance training (RT) on peripheral BDNF levels in individuals with multiple sclerosis (MS). The methods are clearly described, and the interpretation of the findings is appropriate. I have only a few minor comments for the authors to consider:

Comment 1. In the Abstract, line 73, what does “≈80% 1RM” mean? Please also define RM at first use.

REPLY: Although the acronym "1 RM" seemed universally known to those familiar with this type of literature, the question raised by the esteemed reviewer made it evident that this is not the case. Therefore, we have included the full term before introducing the acronym. We thank you for helping us improve the manuscript. For clarification, "1 RM" stands for "one-repetition maximum"; it refers to the load a subject is capable of lifting a single time. We have inserted the sentence below exactly where the esteemed reviewer indicated. From that point onward, we use the previously mentioned acronym "1RM." This insertion is highlighted in yellow in the manuscript. Please see below:

[...] (≈80% of one-repetition maximum: 1RM) [...].

Comment 2. In the Introduction, lines 103–104, if specific recommendations exist regarding the types of exercise used in MS rehabilitation, it would be helpful to describe them explicitly. For example, do the guidelines recommend RT alone or a combination of RT and aerobic exercise?

REPLY: We appreciate the opportunity to clarify this point. Both aerobic and resistance exercises are recommended. Therefore, as suggested, we have added a sentence highlighting this aspect and the position statement recommending both aerobic and resistance exercises. This addition is highlighted in yellow in the manuscript. Please see below:

[...] It is worth noting that both aerobic and resistance exercises are recommended by the position statement on exercise for people with mild to moderate multiple sclerosis from Exercise & Sports Science Australia (ESSA) (Hoang et al., 2022). [...]

Comment 3. In lines 306–315, please provide additional information about the control conditions in the included RCTs. Did participants in the control groups receive no intervention or any specific instructions? This information would complement the description of the interventions.

REPLY: We thank you again for the opportunity to clarify this point. In none of the studies deemed eligible for inclusion in this review and meta-analysis did the individuals in the control group receive any type of intervention. As requested, we have inserted the sentence below to report this information. We would like to emphasize that all changes are highlighted in yellow in the manuscript.

[…] It is worth noting that, in all studies eligible for inclusion in the present study, individuals in the control group did not receive any type of intervention. […]

Comment 4. In Figures 1 and 2, the first group is labelled “BDNF,” whereas this appears to represent the RT intervention group. BDNF is the outcome rather than the intervention, right? Please revise.

REPLY: The reviewer is entirely correct. We have made the change as requested. Please forgive us for this oversight.

Comment 5. Again, in Figures 1 and 2, the labels below the x-axis, “Grupo Controle” and “Favor BNDF”, does not appear accurate. Please revise them to, for example, “Favours control” and “Favours RT.”

REPLY: The reviewer is entirely correct. We have made the change as requested. Please forgive us for this oversight.

REVIEWER #2

This manuscript addresses a relevant and well-defined research question regarding the effects of isolated resistance training on peripheral BDNF levels in individuals with multiple sclerosis. The topic is appropriate for a systematic review and meta-analysis, and the authors should be commended for registering the review protocol, following established systematic review methodologies, and applying recognized tools for risk-of-bias and certainty-of-evidence assessment. Overall, I believe the manuscript has the potential to make a useful contribution to the literature. However, several issues should be addressed before the work can be considered for publication.

Major Comments

Comment 1. Statistical reporting requires clarification and harmonization. The manuscript reports inconsistent pooled effect estimates across different sections. Confidence intervals, test statistics, and p-values associated with the primary meta-analysis should be verified and reported consistently throughout the abstract, results, discussion, tables, and figures. In addition, the manuscript states that a Hartung-Knapp adjustment was used, yet significance testing appears to be reported using Z statistics. The authors should clearly describe the inferential framework used and ensure that all reported results correspond to the final analytical model.

REPLY: Please accept our apologies. The highlighted sections of the text (abstract, statistical analysis, results, and discussion) have undergone extensive revision, and we believe we have corrected the mentioned inconsistencies. Regarding the Hartung–Knapp adjustment, we have inserted the values as requested. Please refer to the excerpts below, which we have added and highlighted in yellow in the manuscript.

Abstract:

[…] With the Hartung–Knapp adjustment, due to the reduced number of studies, the analysis continued to show a positive intervention effect: (SMD=0.49; 95%CI: 0.42 to 0.56; t=3.716; p=0.0339). […]

Statistical Analysis

[…] together with the Hartung–Knapp adjustment (Student’s t – distribution), which is considered more robust for meta-analyses including a small number of studies. The Hartung–Knapp adjustment was applied to account for statistical uncertainty, particularly when few studies are involved (Röver et al., 2015). This adjustment involves modifying the reference distribution by replacing the critical value (Z1−α/2) with the critical value (t1−α/2; k−1) and setting the degrees of freedom to (k−1), where k is the total number of studies included in the meta-analysis (IntHout et al., 2014). Additionally, the standard error is adjusted by multiplying the standard standard error by a correction factor (q), calculated based on Cochrane’s Q statistic and the study weights. […]

[…] The statistical significance of the overall effect was determined using the Z test. In the adjusted analysis, the critical Z-value was replaced by the critical value of the Student's t-distribution, and the degrees of freedom were defined as (k−1), where k is the total number of studies included in the meta-analysis (IntHout et al., 2014). The significance level was set at 5% (p < 0.05). The direction of effects was standardized so that positive SMD values indicated a benefit of the intervention compared to the control group. […]

Results

[…] In addition, with the Hartung–Knapp adjustment, due to the reduced number of studies, the analysis continued to show a positive intervention effect: (SMD=0.49; 95%CI: 0.42 to 0.56; t=3.716; p=0.0339).[…]

Comment 2. Limited evidence base and imprecision. The meta-analysis includes only four studies with a relatively small total sample size. Although the pooled effect suggests a beneficial influence of resistance training on BDNF, the evidence remains limited and imprecise. The conclusions should reflect this uncertainty more explicitly.

REPLY: We agree with the esteemed reviewer. We have rephrased the conclusion and highlighted it in yellow within the manuscript; we believe the current wording addresses the reviewer's request. Please see below:

[…] Furthermore, given that only four studies were included in the meta-analysis, we suggest interpreting the results with some caution. We highlight that the literature still lacks experimental studies analyzing the isolated effect of strength training on BDNF levels in patients with multiple sclerosis.[…]

Comment 3. Clinical and methodological heterogeneity. Included studies differ with respect to disease phenotype, disability status, intervention characteristics, intervention duration, training intensity, and methods used to measure BDNF (serum versus plasma). These differences should be discussed more thoroughly as potential sources of heterogeneity and limitations to interpretation.

REPLY: We appreciate the reviewer's comment; however, we would like to emphasize that, although the studies exhibit the mentioned differences, these were not sufficient to alter the conclusions of the present study. In other words, regardless of these differences, the results indicate that isolated resistance training appears effective in inducing some increase in BDNF levels, even after adjusted analysis. Furthermore, the heterogeneity analysis did not reveal significant variability among the studies. Cochran’s Q test indicated non-significant heterogeneity (Q(3) = 1.681; p = 0.643), and the I² statistic was 0%, with a between-study variance estimate (τ²) of 0.000. These findings suggest that the observed variability in effect sizes is likely attributable to sampling error rather than actual differences between studies, indicating a high degree of consistency in the included evidence. Furthermore, to some extent, it appears we were able to address certain aspects raised by the esteemed reviewer. Please refer to lines 527–579. In that section, we discuss exercise intensity, disease phenotype, clinical status, and the biological matrix used for quantification. Thus, we believe that the manuscript, in its current form, adequately addresses the interpretation of the findings and explicitly highlights the limitations as well as the relative strength of our results.

Comment 4. Data transparency and reproducibility. The Data Availability Statement indicates that data are available within the manuscript and supplementary materials. However, the extraction dataset used to generate the meta-analysis is not clearly available. Providing the complete extraction spreadsheet, calculated effect sizes, and any transformed or estimated values would improve transparency and reproducibility.

REPLY: Please forgive us. There was a mistake on our part. The supplementary material has been made available as requested.

Comment 5. Interpretation of training intensity effects. The suggestion that low- or moderate-intensity resistance training may be more effective than higher-intensity protocols appears to be based on indirect comparisons across a very small number of studies. Similarly, statements regarding potential BDNF-independent mechanisms underlying benefits from higher-intensity training are speculative and should be toned down unless directly supported by the available evidence.

REPLY: We have inserted the sentence below to address the reviewer's request to some extent. It is worth noting that this addition is highlighted in yellow in the manuscript.

[…] On the other hand, due to the limited number of studies, it is not feasible to specify an optimal exercise intensity. Therefore, we suggest caution when interpreting these findings; although some studies suggest a light-to-moderate intensity, given the limited number of experiments, any assertion remains merely speculative. […]

Minor Comments

Comment 6. The manuscript is generally understandable; however, the English language requires revision. Several grammatical errors, typographical mistakes, and awkward expressions are present throughout the text. Professional language editing is recommended.

REPLY: We have carefully revised the entire manuscript to improve the English language, grammar, punctuation, typographical accuracy, and overall readability. Awkward expressions were rewritten to improve clarity and scientific flow, and several minor errors were corrected throughout the text. We believe that the revised version is now clearer and more suitable for publication.

Comment 7. Some terminology and formatting inconsistencies should be corrected to improve clarity and readability.

REPLY: We thank you again for helping to improve the manuscript. We would like to highlight that the manuscript has undergone extensive revision.

Comment 8. The authors should ensure that all supplementary materials, tables, and figure references are correctly labeled and consistently cited in the text.

REPLY: Yes!!

Comment 9. Additional detail regarding data extraction procedures, handling of missing data, and any statistical conversions would strengthen methodological transparency.

REPLY: Thank you. As previously mentioned, we have made a supplementary material table available.

Comment 10. Overall Assessment. The review addresses an important clinical and biological question and applies generally appropriate systematic review and meta-analytic methods. The primary conclusion—that isolated resistance training may increase peripheral BDNF levels in individuals with multiple sclerosis—is broadly supported by the available evidence. However, limitations related to the small evidence base, statistical reporting inconsistencies, data transparency, and interpretation of findings require substantial revision.

REPLY: Thank you for helping us significantly improve the manuscript.

Attachment Submitted filename: Response Letter - MMS 07-07-2026 PLOs ONE.docx

# Peer review: Decision Letter 1

**DOI:** 10.1371/journal.pone.0359019.r003

**Authors:** Tanja Grubić Kezele

14 Aug 2026

<p>PONE-D-26-24021R1

Is Resistance Training Effective to Increase BDNF in Multiple Sclerosis? An Updated Systematic Review and Meta-Analysis of Isolated Interventions

PLOS One

Dear Dr. Cameia,

Thank you for submitting your manuscript to PLOS ONE. After careful consideration, we feel that it has merit but does not fully meet PLOS ONE’s publication criteria as it currently stands. Therefore, we invite you to submit a revised version of the manuscript that addresses the points raised during the review process.

==============================

Your manuscript, entitled "Is Resistance Training Effective to Increase BDNF in Multiple Sclerosis? An Updated Systematic Review and Meta-Analysis of Isolated Interventions", has been reviewed. Your efforts to revise the manuscript are appreciated. However, the peer review process continues because the Reviewer 1 has a one additional comment that the authors should address. Please find the reviewer's comment below.

==============================

Please submit your revised manuscript by Sep 13 2026 11:59PM. If you will need more time than this to complete your revisions, please reply to this message or contact the journal office at plosone@plos.org. When you're ready to submit your revision, log on to https://www.editorialmanager.com/pone/ and select the 'Submissions Needing Revision' folder to locate your manuscript file.

Please include the following items when submitting your revised manuscript:

- A letter that responds to each point raised by the academic editor and reviewer(s). You should upload this letter as a separate file labeled 'Response to Reviewers'.
- A marked-up copy of your manuscript that highlights changes made to the original version. You should upload this as a separate file labeled 'Revised Manuscript with Track Changes'.
- An unmarked version of your revised paper without tracked changes. You should upload this as a separate file labeled 'Manuscript'.

If you would like to make changes to your financial disclosure, please include your updated statement in your cover letter. Guidelines for resubmitting your figure files are available below the reviewer comments at the end of this letter.

If applicable, we recommend that you deposit your laboratory protocols in protocols.io to enhance the reproducibility of your results. Protocols.io assigns your protocol its own identifier (DOI) so that it can be cited independently in the future. For instructions see: https://journals.plos.org/plosone/s/submission-guidelines#loc-laboratory-protocols. Additionally, PLOS ONE offers an option for publishing peer-reviewed Lab Protocol articles, which describe protocols hosted on protocols.io. Read more information on sharing protocols at https://plos.org/protocols?utm_medium=editorial-email&utm_source=authorletters&utm_campaign=protocols.

As the corresponding author, your ORCID iD is verified in the submission system and will appear in the published article. PLOS supports the use of ORCID, and we encourage all coauthors to register for an ORCID iD and use it as well. Please encourage your coauthors to verify their ORCID iD within the submission system before final acceptance, as unverified ORCID iDs will not appear in the published article. *Only*  the individual author can complete the verification step; PLOS staff *cannot*  verify ORCID iDs on behalf of authors.

We look forward to receiving your revised manuscript.

Kind regards,

Tanja Grubić Kezele, Ph.D., M.D.

Academic Editor

PLOS One

**Journal Requirements:**

1. If the reviewer comments include a recommendation to cite specific previously published works, please review and evaluate these publications to determine whether they are relevant and should be cited. There is no requirement to cite these works unless the editor has indicated otherwise.

2. Please review your reference list to ensure that it is complete and correct. If you have cited papers that have been retracted, please include the rationale for doing so in the manuscript text, or remove these references and replace them with relevant current references. Any changes to the reference list should be mentioned in the rebuttal letter that accompanies your revised manuscript. If you need to cite a retracted article, indicate the article’s retracted status in the References list and also include a citation and full reference for the retraction notice.

[Note: HTML markup is below. Please do not edit.]

Reviewers' comments:

Reviewer's Responses to Questions

**Comments to the Author**

1. If the authors have adequately addressed your comments raised in a previous round of review and you feel that this manuscript is now acceptable for publication, you may indicate that here to bypass the “Comments to the Author” section, enter your conflict of interest statement in the “Confidential to Editor” section, and submit your "Accept" recommendation.

Reviewer #1: (No Response)

**********

2. Is the manuscript technically sound, and do the data support the conclusions?

The manuscript must describe a technically sound piece of scientific research with data that supports the conclusions. Experiments must have been conducted rigorously, with appropriate controls, replication, and sample sizes. The conclusions must be drawn appropriately based on the data presented.

Reviewer #1: Yes

**********

3. Has the statistical analysis been performed appropriately and rigorously?

Reviewer #1: Yes

**********

4. Have the authors made all data underlying the findings in their manuscript fully available?

The PLOS Data policy requires authors to make all data underlying the findings described in their manuscript fully available without restriction, with rare exception (please refer to the Data Availability Statement in the manuscript PDF file). The data should be provided as part of the manuscript or its supporting information, or deposited to a public repository. For example, in addition to summary statistics, the data points behind means, medians and variance measures should be available. If there are restrictions on publicly sharing data—e.g. participant privacy or use of data from a third party—those must be specified.

Reviewer #1: Yes

**********

5. Is the manuscript presented in an intelligible fashion and written in standard English?

PLOS ONE does not copyedit accepted manuscripts, so the language in submitted articles must be clear, correct, and unambiguous. Any typographical or grammatical errors should be corrected at revision, so please note any specific errors here.

Reviewer #1: Yes

**********

6. Review Comments to the Author

Please use the space provided to explain your answers to the questions above. You may also include additional comments for the author, including concerns about dual publication, research ethics, or publication ethics. (Please upload your review as an attachment if it exceeds 20,000 characters)

Reviewer #1: In Figure 1 and Figure 2, the group heading is still BNDF and CONTROL. I understand the mean and SD values shown are the BNDF values. The group heading should be something like Intervention group and Control group, rather than BNDF. Please correct.

**********

7. PLOS authors have the option to publish the peer review history of their article (what does this mean?). If published, this will include your full peer review and any attached files.

If you choose “no”, your identity will remain anonymous but your review may still be made public.

**Do you want your identity to be public for this peer review?**  For information about this choice, including consent withdrawal, please see our Privacy Policy.

Reviewer #1: No

**********

[NOTE: If reviewer comments were submitted as an attachment file, they will be attached to this email and accessible via the submission site. Please log into your account, locate the manuscript record, and check for the action link "View Attachments". If this link does not appear, there are no attachment files.]

To ensure your figures meet our technical requirements, please review our figure guidelines: https://journals.plos.org/plosone/s/figures

You may also use PLOS’s free figure tool, NAAS, to help you prepare publication quality figures: https://journals.plos.org/plosone/s/figures#loc-tools-for-figure-preparation.

NAAS will assess whether your figures meet our technical requirements by comparing each figure against our figure specifications.

# Author comment: Author response to Decision Letter 2

**DOI:** 10.1371/journal.pone.0359019.r004

20 Aug 2026

PONE-D-26-24021R1

Is Resistance Training Effective to Increase BDNF in Multiple Sclerosis? An Updated Systematic Review and Meta-Analysis of Isolated Interventions

PLOS One

Journal Requirements:

Comment 1. If the reviewer comments include a recommendation to cite specific previously published works, please review and evaluate these publications to determine whether they are relevant and should be cited. There is no requirement to cite these works unless the editor has indicated otherwise.

REPLY: OK, understood. The reviewers did not require the inclusion of any out-of-scope references.

Comment 2. Please review your reference list to ensure that it is complete and correct. If you have cited papers that have been retracted, please include the rationale for doing so in the manuscript text, or remove these references and replace them with relevant current references. Any changes to the reference list should be mentioned in the rebuttal letter that accompanies your revised manuscript. If you need to cite a retracted article, indicate the article’s retracted status in the References list and also include a citation and full reference for the retraction notice.

REPLY: All references were included using EndNote software. All are relevant to the study. No references have been retracted.

Reviewer's Responses to Questions

Comments to the Author

Reviewer #1: In Figure 1 and Figure 2, the group heading is still BNDF and CONTROL. I understand the mean and SD values shown are the BNDF values. The group heading should be something like Intervention group and Control group, rather than BNDF. Please correct.

REPLY: We apologize for the error. The reviewer is indeed correct. We had previously adjusted the figure but overlooked other important elements. Consider it done. Observe the adjusted figures below.

Attachment Submitted filename: RESPONSE LETTER_R2.docx

# Peer review: Decision Letter 2

**DOI:** 10.1371/journal.pone.0359019.r005

**Authors:** Tanja Grubić Kezele

8 Sep 2026

Is Resistance Training Effective to Increase BDNF in Multiple Sclerosis? An Updated Systematic Review and Meta-Analysis of Isolated Interventions

PONE-D-26-24021R2

Dear Dr. Cameia,

We’re pleased to inform you that your manuscript has been judged scientifically suitable for publication and will be formally accepted for publication once it meets all outstanding technical requirements.

Within one week, you’ll receive an e-mail detailing the required amendments. When these have been addressed, you’ll receive a formal acceptance letter and your manuscript will be scheduled for publication.

An invoice will be generated when your article is formally accepted. Please note, if your institution has a publishing partnership with PLOS and your article meets the relevant criteria, all or part of your publication costs will be covered. Please make sure your user information is up-to-date by logging into Editorial Manager at Editorial Manager® and clicking the ‘Update My Information' link at the top of the page. For questions related to billing, please contact billing support.

If your institution or institutions have a press office, please notify them about your upcoming paper to help maximize its impact. If they’ll be preparing press materials, please inform our press team as soon as possible -- no later than 48 hours after receiving the formal acceptance. Your manuscript will remain under strict press embargo until 2 pm Eastern Time on the date of publication. For more information, please contact onepress@plos.org.

Kind regards,

Tanja Grubić Kezele, Ph.D., M.D.

Academic Editor

PLOS One

Additional Editor Comments (optional):

Reviewers' comments:

Reviewer's Responses to Questions

**Comments to the Author**

Reviewer #1: All comments have been addressed

**********

2. Is the manuscript technically sound, and do the data support the conclusions?

Reviewer #1: (No Response)

**********

3. Has the statistical analysis been performed appropriately and rigorously?

Reviewer #1: (No Response)

**********

4. Have the authors made all data underlying the findings in their manuscript fully available?

The PLOS Data policy

Reviewer #1: (No Response)

**********

5. Is the manuscript presented in an intelligible fashion and written in standard English?

Reviewer #1: (No Response)

**********

Reviewer #1: (No Response)

**********

what does this mean?). If published, this will include your full peer review and any attached files.

If you choose “no”, your identity will remain anonymous but your review may still be made public.

**Do you want your identity to be public for this peer review?** For information about this choice, including consent withdrawal, please see our Privacy Policy

Reviewer #1: No

**********

# Editor report: Acceptance letter

**DOI:** 10.1371/journal.pone.0359019.r006

**Authors:** Tanja Grubić Kezele

PONE-D-26-24021R2

PLOS One

Dear Dr. Cameia,

I'm pleased to inform you that your manuscript has been deemed suitable for publication in PLOS One. Congratulations! Your manuscript is now being handed over to our production team.

At this stage, our production department will prepare your paper for publication. This includes ensuring the following:

* All references, tables, and figures are properly cited

* All relevant supporting information is included in the manuscript submission,

* There are no issues that prevent the paper from being properly typeset

You will receive further instructions from the production team, including instructions on how to review your proof when it is ready. Please keep in mind that we are working through a large volume of accepted articles, so please give us a few days to review your paper and let you know the next and final steps.

Lastly, if your institution or institutions have a press office, please let them know about your upcoming paper now to help maximize its impact. If they'll be preparing press materials, please inform our press team within the next 48 hours. Your manuscript will remain under strict press embargo until 2 pm Eastern Time on the date of publication. For more information, please contact onepress@plos.org.

You will receive an invoice from PLOS for your publication fee after your manuscript has reached the completed accept phase. If you receive an email requesting payment before acceptance or for any other service, this may be a phishing scheme. Learn how to identify phishing emails and protect your accounts at https://explore.plos.org/phishing.

If we can help with anything else, please email us at customercare@plos.org.

Thank you for submitting your work to PLOS One and supporting open access.

Kind regards,

PLOS One Editorial Office Staff

on behalf of

Prof. dr. Tanja Grubić Kezele

Academic Editor

PLOS One
